# Senescent Microglia Mediate Neuroinflammation‐Induced Cognitive Dysfunction by Selective Elimination of Excitatory Synapses in the Hippocampal CA1

**DOI:** 10.1111/acel.70167

**Published:** 2025-07-07

**Authors:** Kai Liu, Di Fan, Hai‐peng Wu, Xiao‐yi Hu, Qiu‐li He, Xin‐miao Wu, Cui‐na Shi, Jian‐jun Yang, Mu‐huo Ji

**Affiliations:** ^1^ Department of Anesthesiology The Second Affiliated Hospital of Nanjing Medical University Nanjing China; ^2^ Department of Anesthesiology, Pain and Perioperative Medicine The First Affiliated Hospital of Zhengzhou University Zhengzhou China

**Keywords:** long‐term potentiation, microglia, neuroinflammation, senescence, synaptic plasticity

## Abstract

Microglia‐mediated neuroinflammation has been shown to exert an important effect on the progression of a growing number of neurodegenerative disorders. Prolonged exposure to detrimental stimuli leads to a state of progressive activation and aging‐related features in microglia (also termed as senescent microglia). However, the mechanisms by which senescent microglia contribute to neuroinflammation‐induced cognitive dysfunction remain to be elucidated. Here, we developed a mouse model of neuroinflammation induced by lipopolysaccharides at 0.5 mg/kg for 7 consecutive days. To evaluate cognitive function, C57BL/6J mice were employed and subjected to a series of behavioral assessments, including the open field, Y‐maze, and novel object recognition tests. Employing single‐cell RNA sequencing technology, we have delved into the differential expressions of RNA within microglia. Furthermore, to investigate anatomic and physiological alterations of pyramidal neurons, we utilized Golgi staining and whole‐cell patch‐clamp recordings, respectively. Validation of our results in protein expression was performed using western blotting and immunofluorescence. We specifically identified senescent microglia with a high expression of p16^INK4a^ and observed that microglia in the hippocampal CA1 region of the model exhibited signatures of elevated phagocytosis and senescence. A senolytic by ABT‐737 treatment alleviated the production of senescence‐associated secretory phenotypes, the accumulation of senescent microglia, and the microglial hyperphagocytosis of excitatory synapses following LPS exposures. This treatment also restored reduced excitatory synaptic transmission, impaired long‐term potentiation, and cognitive function in the model. These results indicate that reducing senescent microglia may potentially serve as a therapeutic approach to prevent neuroinflammation‐related cognitive dysfunction.

## Graphical abstract

In the CA1, microglia are activated to be responsible for synaptic pruning. However, LPS induces a senescent phenotype in microglia, characterized by the elevated levels of p16, as well as the production of SASPs. These senescent microglia mediate the removal of excitatory synapses selectively, resulting in dendritic spine loss and LTP impairment, which ultimately contribute to cognitive dysfunction in mice.

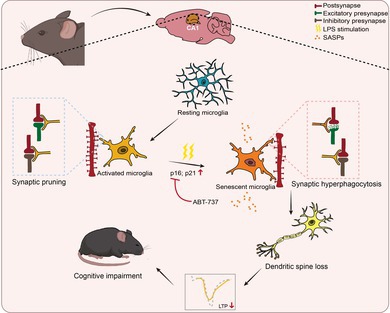

## 1 Introduction

Neuroinflammation refers to the immune system's response to injury or disease within the nervous system, which is crucial in the initiation and progression of numerous neurological disorders, especially neurodegenerative diseases like Alzheimer's disease (AD) (Leng and Edison 2021). Prolonged or excessive neuroinflammatory responses can result in neurotoxicity, which may lead to cerebral damage and facilitate the emergence and progression of multiple brain pathologies. Previous reports have proven that neuroinflammation is a contributing factor in a range of diseases associated with cognitive dysfunction (Leng and Edison 2021; Isik et al. 2023; Ravizza et al. 2024). However, the precise mechanisms are still unknown.

Microglia, the resident macrophages in the central nervous system (CNS), play a leading role in the regulation of neuroinflammation and the maintenance of homeostatic balance during the physiological state (Leng and Edison 2021). Typically, microglia exhibit dual roles: they are involved in both the promotion of neuroinflammation and the associated pathological processes, as well as in the resolution of neuroinflammation and the repair of the CNS (Woodburn et al. 2021). This underscores the complexities of neuroinflammation and microglial phenotypes in the context of disease. Additionally, microglia‐mediated synaptic pruning serves as a crucial mechanism for the refinement of neural circuits during brain development (Colonna and Butovsky 2017; Bohlen et al. 2019). In the mature CNS, synaptic pruning by microglia facilitates synaptic turnover, removes unnecessary synapses, and contributes to the formation of previously non‐existent neuronal circuits (Colonna and Butovsky 2017). Conversely, under pathological conditions, dysregulated synaptic pruning by microglia can underlie the mechanisms of various neurodegenerative diseases. A previous study unveiled that microglial enhanced engulfment of C1q‐tagged synapses plays a critical role in the pathogenesis of sepsis‐associated encephalopathy (Chung et al. 2023). Therefore, maintaining synaptic homeostasis may represent a viable therapeutic strategy for neuroinflammatory disorders.

Cellular senescence is characterized by a stable cessation of growth, accompanied by a hypersecretory and pro‐inflammatory phenotype, collectively referred to as senescence‐associated secretory phenotypes (SASPs) (de Magalhaes 2024). This phenomenon is a critical biological process wherein cells enter an irreversible growth arrest following a phase of proliferation or in response to sublethal stressors or oncogene activation (de Magalhaes 2024; Melo et al. 2024). Besides, senescence is intricately linked to the upregulation of cyclin‐dependent kinase (CDK) inhibitors, particularly CDKN2A/p16^Ink4a^ (p16) (Reyes et al. 2022) and CDKN1A/p21^Waf1/Cip1^ (p21) (Yao et al. 2023). This stasis in cell cycle progression is accompanied by notable alterations in cellular morphology and architecture. Senescent cells typically exhibit an augmented cellular volume and enhanced lysosomal activity, as evidenced by the pronounced expression of senescence‐associated β‐galactosidase (SA‐β‐gal) (Melo et al. 2024). Microglia, due to their highly dynamic nature and elevated proliferative capacity, exhibit increased susceptibility to pathological damage and acquired dysfunction under neurodegenerative conditions (Hu et al. 2021). Nevertheless, the identification of definitive markers of senescent microglia poses a significant challenge. For example, a recent research indicated that senescent microglia display a distinct signature that differentiates them from disease‐associated microglia, despite both subtypes exhibiting high levels of triggering receptor expressed on myeloid cells‐2 (TREM2) (Rachmian et al. 2024).

Here, we aimed to investigate the potential role of senescent microglia in the hippocampal CA1 region of the mouse model of neuroinflammation, and explore the relationship between microglial senescence and synaptic disequilibrium. In this study, we identified senescent microglia with high expression of p16 in the neuroinflammation model induced by lipopolysaccharides (LPS). Senolytic treatment with ABT‐737, a Bcl‐2 family inhibitor (Kolodkin‐Gal et al. 2022; Rachmian et al. 2024), alleviated senescent microglial phagocytosis of excitatory synapses in the CA1, leading to the restoration of dendritic spines and synaptic transmission, ultimately resulting in cognitive improvement in mice after LPS exposures.

## 2 Results

### 2.1 LPS‐Induced Neuroinflammation Led to Increased Expression of Lysosomal and Aging Related Genes in Microglia

**FIGURE 1:**
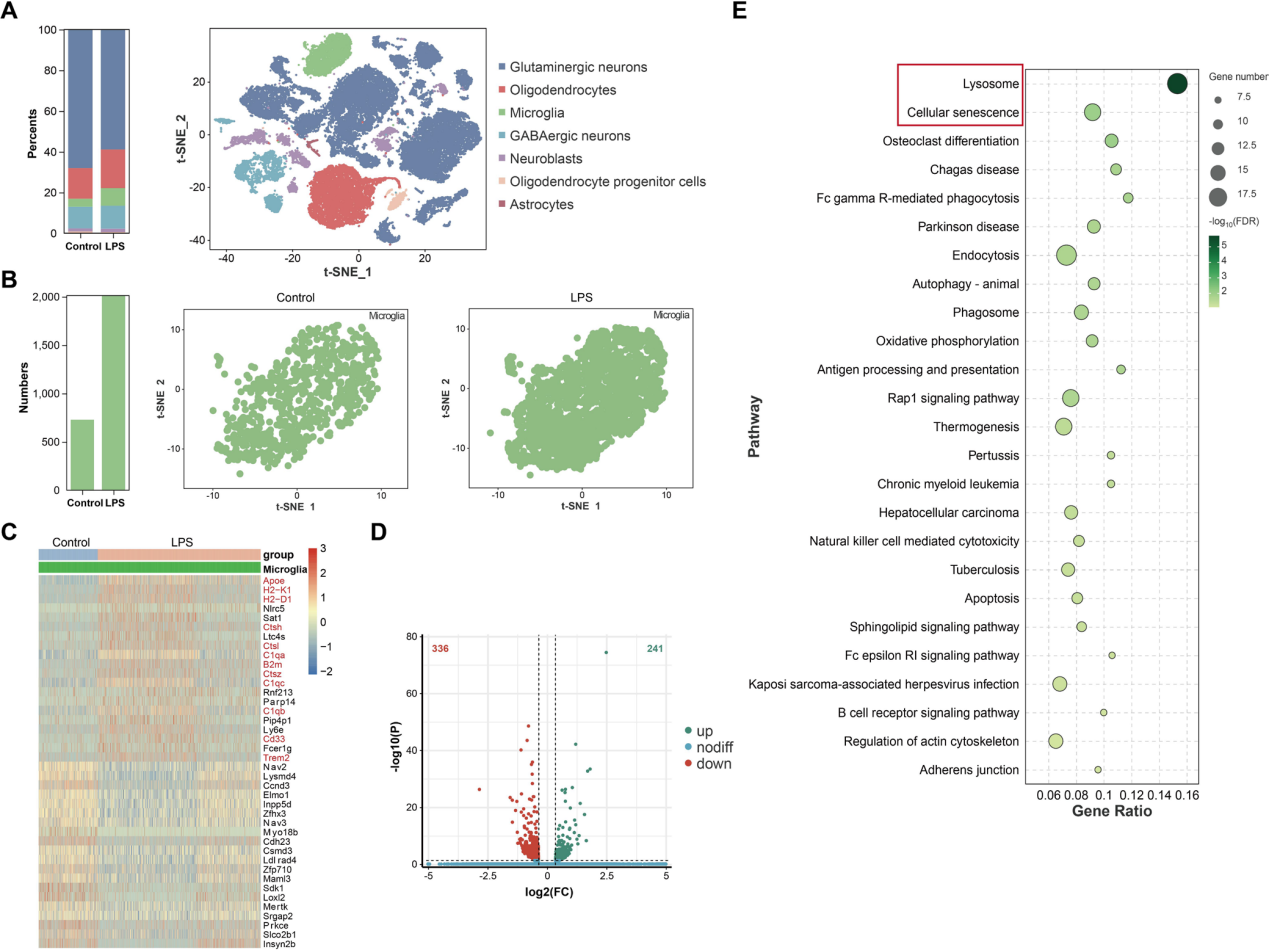
LPS‐induced neuroinflammation led to increased expression of lysosomal and aging related genes in microglia. (A) Percent of cell clusters between the control and LPS groups, and the t‐SNE plot of 42,069 cells in the hippocampi from C57BL/6 mice. Each data point represents one cell, with different colored clusters by cell type assignments. *N* = 2 biological replicates for each group. (B) Percent of microglia clusters between the control and LPS groups, and the t‐SNE plot of microglia clusters in the hippocampi from C57BL/6 mice. Each data point represents one cell. *N* = 2 biological replicates for each group. (C) The heat map of DEGs in microglia between the control and LPS groups. (D) The volcano plot with DEGs in microglia between the control and LPS groups. (E) The top 25 KEGG pathways enriched in microglia between the control and LPS groups.

To investigate the potential mechanisms underlying neuroinflammation induced by LPS, we employed single‐cell RNA sequencing (scRNA‐seq) to analyze the cell repertoires of the hippocampi obtained from 8 to 10 weeks old C57BL/6 mice in both the control and LPS groups. We identified distinct cell clusters based on specific sets of marker genes, which facilitated their annotation and elucidated the major subsets present within the samples (Figure 1A). Subsequently, we found the percent of the microglia cluster in the LPS group was significantly increased in comparison to the control group (Figure 1B). Thereby, we identified this microglia cluster for comparative gene analysis between the control and LPS groups. The Volcano plot showed a total of 241 up‐regulated and 336 down‐regulated genes in the LPS group compared with the control group (Figure 1D). Interestingly, among the top 20 up‐regulated genes, eight specific genes (*ctsh*, *ctsl*, *c1qa*, *ctsz*, *c1qc*, *c1qb*, *cd33*, and *trem2*) were linked to microglial phagocytosis (Cai et al. 2019; Dejanovic et al. 2022; Jiang et al. 2022; Li et al. 2023; Eskandari‐Sedighi et al. 2024), while five genes (*apoe*, *H2‐D1*, *H2‐K1*, *b2m*, and *trem2*) were associated with cellular senescence (Zhao et al. 2022; Kellogg et al. 2023; Rachmian et al. 2024) (Figure 1C). Consistently, the KEEG enrichment analysis indicated that the lysosomal pathways, associated with the process of microglial phagocytosis, as well as cellular senescence pathways, linked to the decline in proliferation, differentiation, and physiological functions, exhibited significant relevance within the microglia cluster (Figure 1E).

### 2.2 LPS Increased Microglial Phagocytosis and Senescent Microglia in the CA1

**FIGURE 2:**
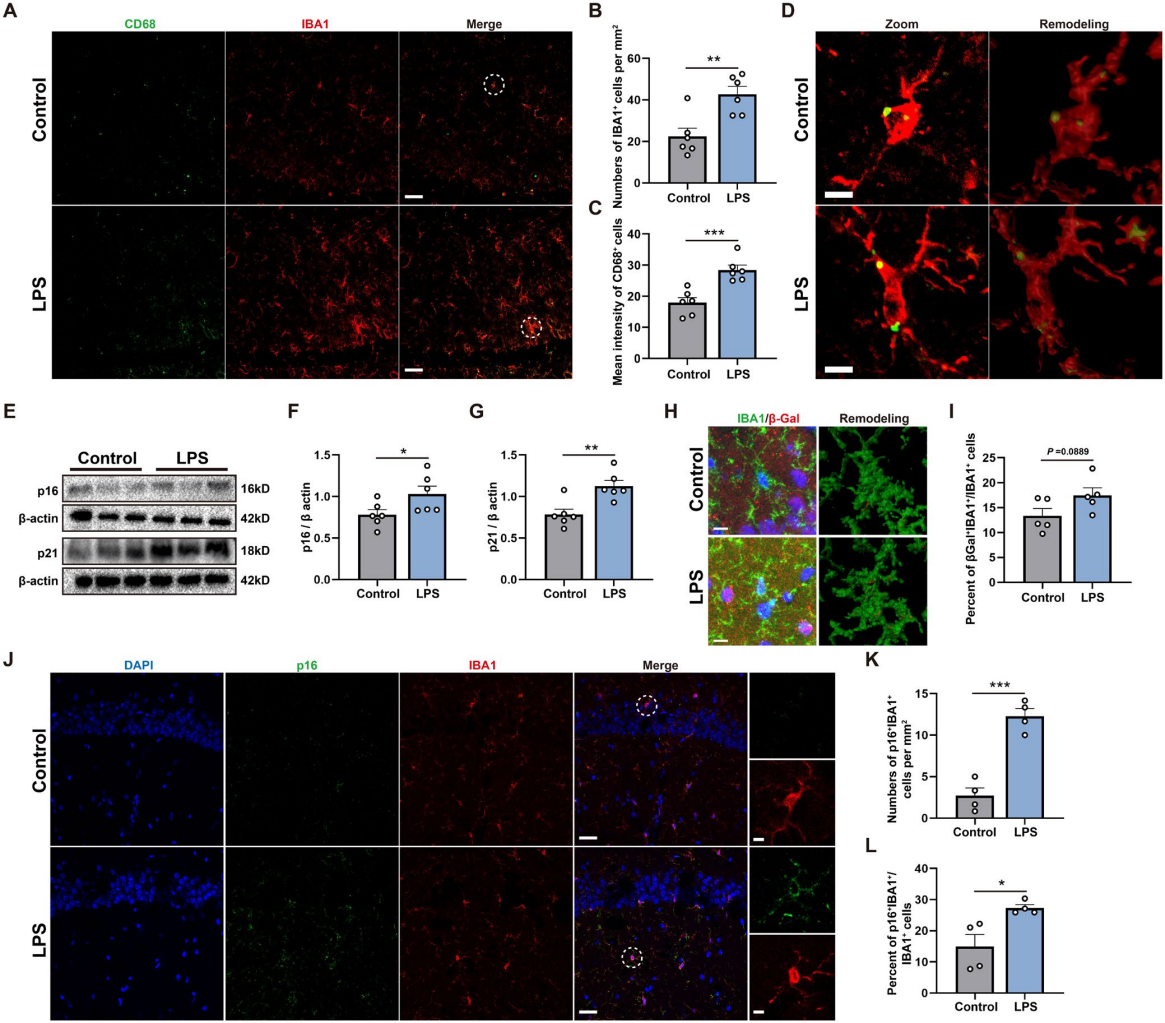
LPS increased microglial phagocytosis and senescent microglia in the CA1. (A) Representative images immunostained for CD68^+^ (green) and IBA1^+^ (red) in the hippocampal CA1 between the control and LPS groups. Scale bar = 20 μm. (B) The number of IBA1^+^ cells in the hippocampal CA1 between the control and LPS groups, *N* = 6 mice for each group. (C) The intensity of CD68^+^ cells in the hippocampal CA1 between the control and LPS groups, *N* = 6 mice for each group. (D) 3D reconstruction of phagocytic activity in microglia. Scale bar = 4 μm. (E) Quantification of normalized p16 and p21 protein intensities. (F) Relative expression of p16/β actin in the hippocampi between the control and LPS groups, *N* = 6 mice for each group. (G) Relative expression of p21/β actin in the hippocampi between the control and LPS groups, *N* = 6 mice for each group. (H) Representative images immunostained for IBA1^+^ (green) and β‐Gal^+^ (red) and 3D reconstruction in the hippocampal CA1 between the control and LPS groups. Scale bar = 4 μm. (I) The percent of β‐Gal^+^IBA1^+^ cells in the hippocampal CA1 between the control and LPS groups, *N* = 5 mice for each group. (J) Representative images immunostained for DAPI^+^ (blue), p16^+^ (green) and IBA1^+^ (red) in the hippocampal CA1 between the control and LPS groups. Scale bar = 20 μm, Zoom = 4 μm. (K) The number of p16^+^IBA1^+^ cells in the hippocampal CA1 between the control and LPS groups, *N* = 4 mice for each group. (L) The percent of p16^+^IBA1^+^ cells in the hippocampal CA1 between the control and LPS groups, *N* = 4 mice for each group. All values are shown as mean ± SEM by two‐tailed *t* test, *p < 0.05, **p < 0.01, ***p < 0.001.

To examine the microglial proliferation and phagocytosis after LPS exposures, we measured the number of IBA1^+^ cells (a marker of microglia) and the mean intensity of CD68^+^ cells (a marker of lysosome). In comparison to the control group, there was a notable increase in the number of IBA1^+^ cells, and the phagocytic activity of microglia was significantly elevated in the CA1 of the LPS group (Figure 2A,B, *t* = 3.745, *p* = 0.0038; Figure 2C, *t* = 4.618, *p* = 0.0010). 3D reconstruction was performed in Figure 2D. To ascertain whether exposure to LPS induces microglial senescence, we assessed the expression level of p16 and p21, two classic biomarkers of cellular senescence (de Magalhaes 2024). Compared with the control group, the notable elevation of p16 and p21 was found in the hippocampi of mice in the LPS group (Figure 2E,F, *t* = 2.245, *p* = 0.0486; Figure 2G, *t* = 3.690, *p* = 0.0042). Moreover, SA‐β‐gal activity was also detected, which is a measure of lysosomal galactosidase activity at pH 6.0 and widely used as a biomarker of senescent cells both in vitro and in vivo (Dungan et al. 2022; de Magalhaes 2024). However, there was no significant difference in the percent of β‐gal^+^IBA1^+^ cells between the control and LPS groups (Figure 2H,I, *t* = 1.936, *p* = 0.0889). Additionally, the number and percent of co‐labeled IBA1^+^ and p21^+^ cells in the CA1 between the control and LPS groups also had no statistically significant difference (Figure S1A,B, *t* = 1.000, *p* = 0.3559; Figure S1C, *t* = 0.2608, *p* = 0.8030). Nevertheless, the number and percent of co‐labeled IBA1^+^ and p16^+^ cells increased significantly in the CA1 of the LPS group compared with the control group (Figure 2J,K, *t* = 7.335, *p* = 0.0003; Figure 2L, *t* = 3.081, *p* = 0.0216). These results suggested that senescent microglia expressing p16 but not p21 can be induced by repeated LPS exposures.

In order to eliminate the potential influence of senescent neurons and other glial cells, we conducted co‐localization of p16 staining with neuronal (NEUN) and astrocytic markers (GFAP) in the CA1. The results revealed no significant alteration in the percent of p16^+^NEUN^+^ and p16^+^GFAP^+^ cells; however, an increase in the number of GFAP^+^ cells was observed in the LPS group (Figure S2A,B, *t* = 3.422, *p* = 0.0141; Figure S2C, *t* = 0.7712, *p* = 0.4699; Figure S2D,E, *t* = 0.2159, *p* = 0.8362; Figure S2F, *t* = 0.7597, *p* = 0.4762).

In the murine brain, the hippocampus and medial prefrontal cortex (mPFC) constitute pivotal neural substrates regulating cognitive processing (Griffin 2021). To rule out potential influences from other subregions, we focused on investigating glial cell responses within the dentate gyrus (DG) and the prelimbic cortex (PrL) of the mPFC. Specifically, we conducted analyses of astrocytic and microglial activation within the DG and the PrL by immunofluorescence. It showed that the number of IBA1^+^ cells and GFAP^+^ cells in the PrL was significantly increased following LPS exposures compared with the control group (Figure S3A,B, *t* = 5.621, *p* = 0.0014; Figure S3C, *t* = 20.33, *p* < 0.0001). However, we did not observe significant change of senescent microglia (p16^+^IBA1^+^ cells) induced by LPS in the PrL (Figure S3D,E, *t* = 0.8218, *p* = 0.4426). In addition, in the DG, there was no obvious change in the number of IBA1^+^ cells between the control and LPS groups. Compared with the control group, the number of GFAP^+^ cells was remarkably enhanced by LPS. However, this was not the primary purpose of our investigation (Figure S3F,G, *t* = 0.1111, *p* = 0.9152; Figure S3H, *t* = 6.072, *p* = 0.0009).

### 2.3 LPS Increased Microglial Phagocytosis of Excitatory but Not Inhibitory Synapses in the CA1

**FIGURE 3:**
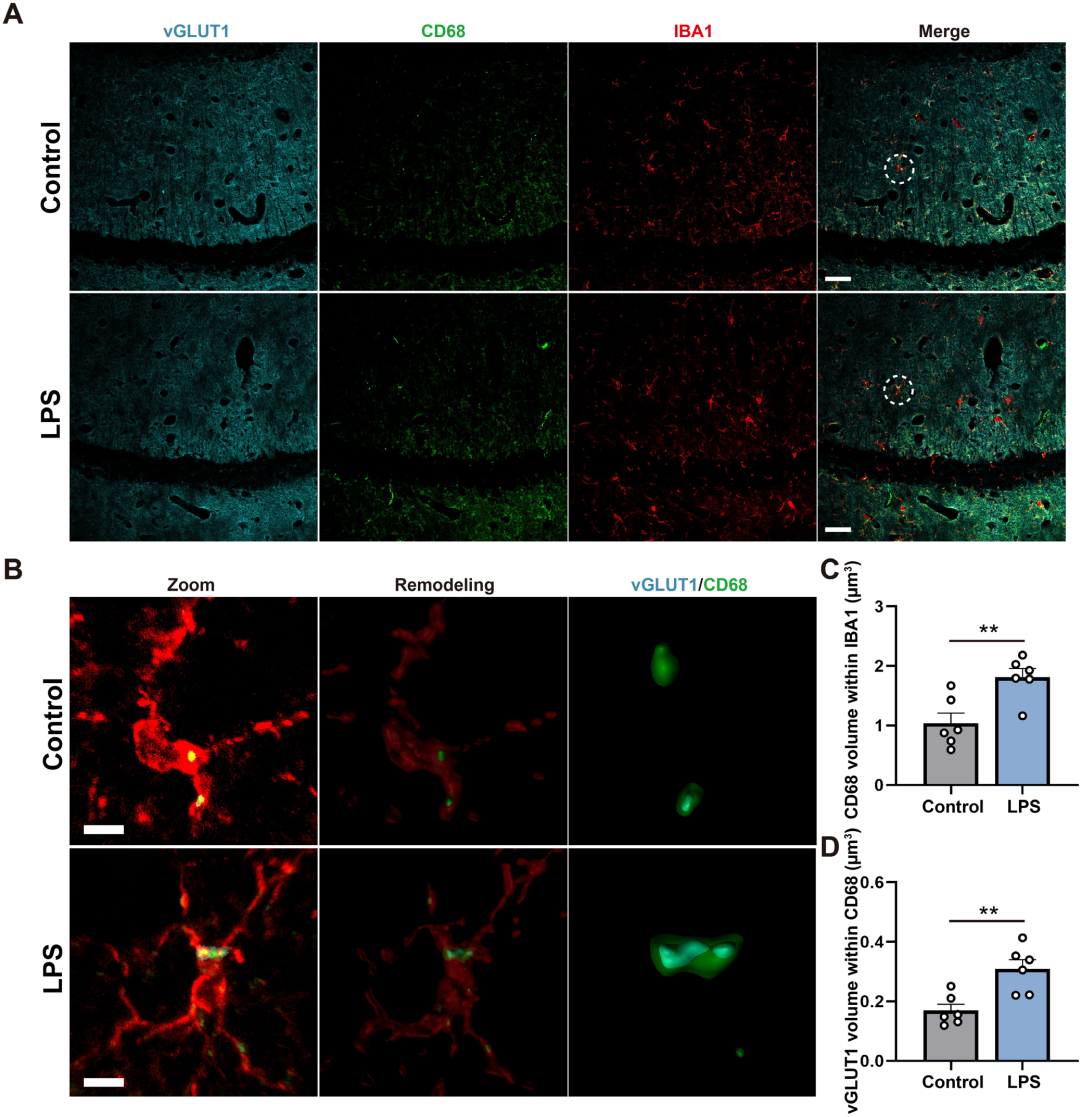
LPS increased microglial phagocytosis of excitatory synapses in the CA1. (A) Representative images immunostained for vGLUT1^+^ (blue), CD68^+^ (green) and IBA1^+^ (red) in the hippocampal CA1 between the control and LPS groups. Scale bar = 20 μm. (B) Representative images immunostained for vGLUT1^+^ (blue), CD68^+^ (green) and IBA1^+^ (red) in Zoom and for 3D reconstruction. Scale bar = 4 μm. (C) Quantification of CD68 volume within IBA1 volume in the hippocampal CA1 between the control and LPS groups, *N* = 6 mice for each group. (D) Quantification of vGLUT1 volume within CD68 volume in the hippocampal CA1 between the control and LPS groups, *N* = 6 mice for each group. All values are shown as mean ± SEM by two‐tailed *t* test, **p < 0.01.

**FIGURE 4:**
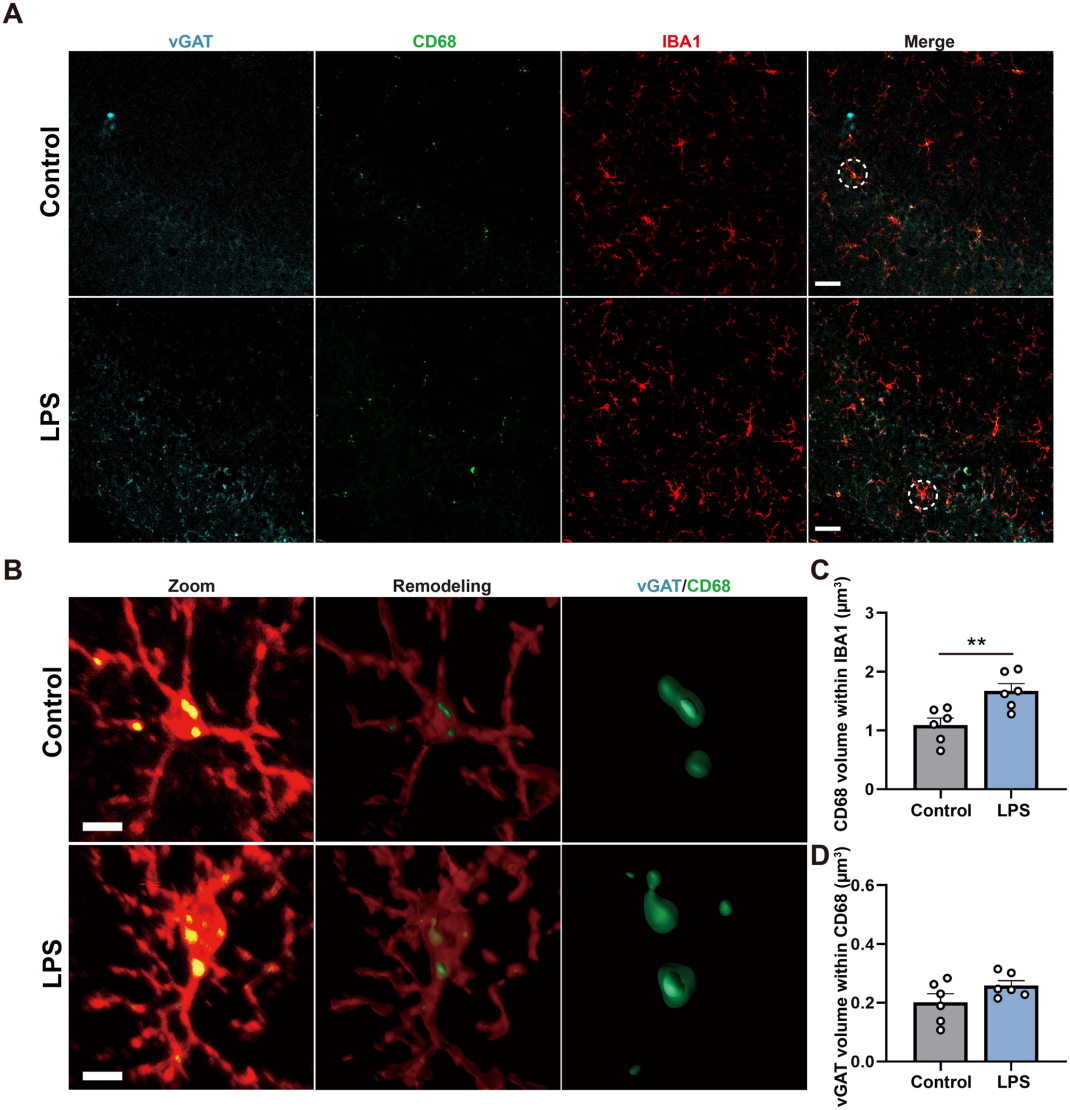
LPS did not alter microglial phagocytosis of inhibitory synapses in the CA1. (A) Representative images immunostained for vGAT^+^ (blue), CD68^+^ (green) and IBA1^+^ (red) in the hippocampal CA1 between the control and LPS groups. Scale bar = 20 μm. (B) Representative images immunostained for vGAT^+^ (blue), CD68^+^ (green) and IBA1^+^ (red) in Zoom and for 3D reconstruction. Scale bar = 4 μm. (C) Quantification of CD68 volume within IBA1 volume in the hippocampal CA1 between the control and LPS groups, *N* = 6 mice for each group. (D) Quantification of vGAT volume within CD68 volume in the hippocampal CA1 between the control and LPS groups, *N* = 6 mice for each group. All values are shown as mean ± SEM by two‐tailed *t* test, **p < 0.01.

Accumulating evidence suggests that the complement system can modulate synaptic connectivity and cognitive function through synaptic pruning during normal development, adulthood, and aging (Gomez‐Arboledas et al. 2021; Li et al. 2024). Our results showed that complement‐related genes of microglia (*c1qa*, *c1qb*, *c1qc*) after LPS treatment were up‐regulated (Figure 1C). As an important component of the innate immune system, complement C1q plays a key role in microglia‐mediated synaptic pruning (Chung et al. 2023; Li et al. 2024). Therefore, we measured the microglial phagocytosis of excitatory and inhibitory synapses by immunofluorescence and 3D reconstruction analysis. The images and 3D reconstructions of co‐labeled IBA1^+^, CD68,^+^ and vGLUT1^+^ cells were shown in Figure 3A,B, and IBA1^+^, CD68,^+^ and vGAT^+^ cells were shown in Figure 4A,B. The results showed that the microglial CD68 volume and the volume of vGLUT1 internalized within microglial CD68 both increased markedly in the LPS group compared with the control group (Figure 3C, *t* = 3.468, *p* = 0.0060; Figure 3D, *t* = 3.730, *p* = 0.0039). Nevertheless, there was no significant difference in the volume of vGAT internalized within microglial CD68 between the control and LPS groups (Figure 4C, *t* = 3.406, *p* = 0.0067; Figure 4D, *t* = 1.712, *p* = 0.1177). These results indicated that LPS may selectively accelerate microglial phagocytosis of excitatory synapses. However, whether microglial senescence was associated with microglial hyperphagocytosis remains unclear.

### 2.4 Senolytic Therapy Improved LPS‐Induced Cognitive Dysfunction in Mice

**FIGURE 5:**
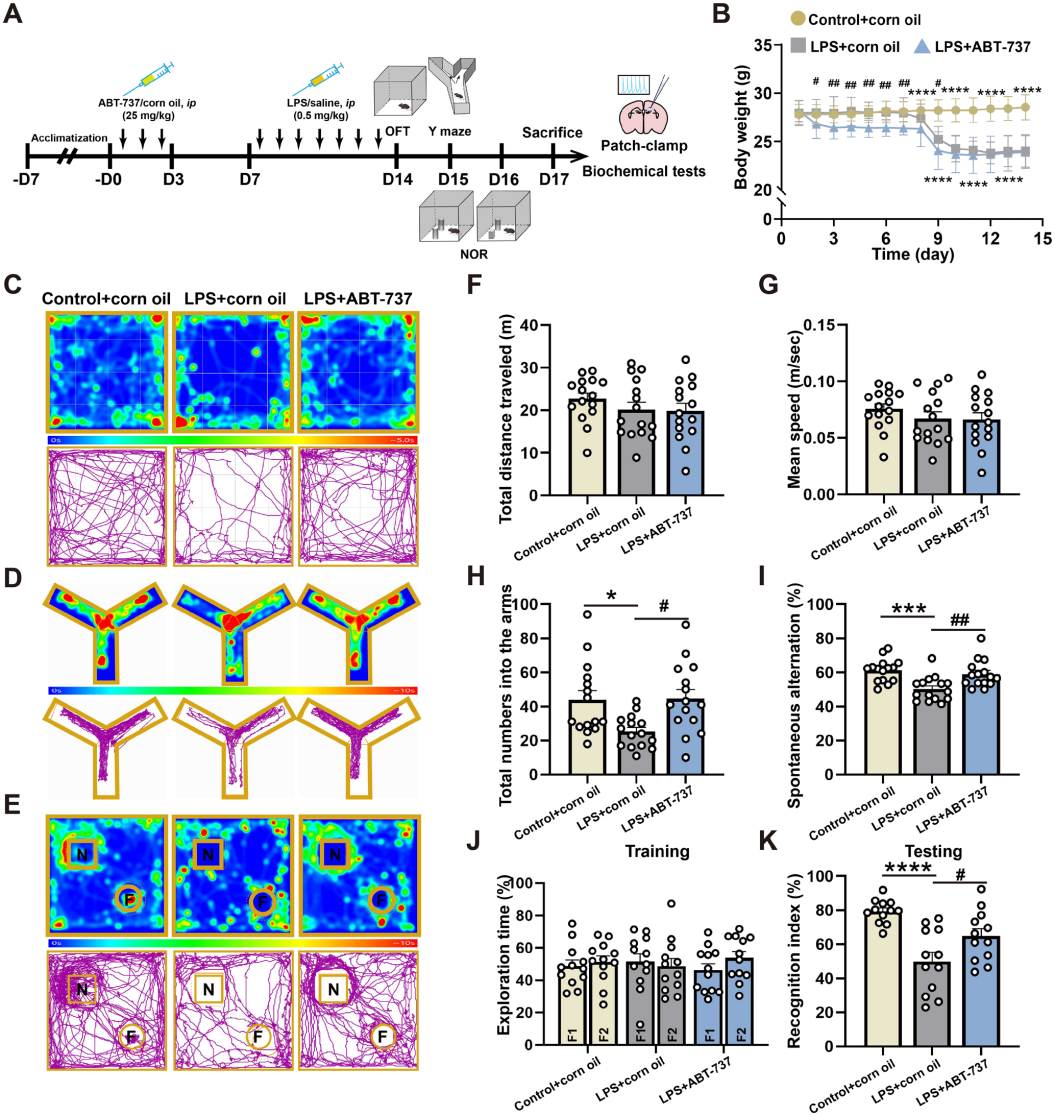
Senolytic therapy improved LPS‐induced cognitive dysfunction in mice. (A) Schematic representation of the experimental procedures. (B) Variations in the body weight of mice throughout the experimental procedure, *N* = 15 mice for each group. (C) Representative heat maps and track charts in the OFT. (D) Representative heat maps and track charts in the Y‐maze test. (E) Representative heat maps and track charts in the NOR test. (F) Total distance in the OFT among experimental groups, *N* = 15 mice for each group. (G) Mean speed in the OFT among experimental groups, *N* = 15 mice for each group. (H) Total number into the arms in the Y‐maze test among experimental groups, *N* = 15 mice for each group. (I) Spontaneous alteration in the Y‐maze test among experimental groups, *N* = 15 mice for each group. (J) The proportion of exploring for F1 and F2 in the NOR test among experimental groups, *N* = 15 mice for each group. (K) Recognition index of exploring for familiar object (F) and novel object (N) in the NOR test among experimental groups, *N* = 15 mice for each group. All values are shown as mean ± SEM by one‐way ANOVA with Tukey's post hoc test, *p < 0.05, ***p < 0.001, ****p < 0.0001, ^#^ *p* < 0.05, ^##^ *p* < 0.01.

Our previous study showed that the LPS treatment for 7 days can induce cognitive dysfunction in mice. The timeline of behavior assessments including the open field test (OFT), Y‐maze, and novel object recognition tests (NOR) was also described in Figure 5A. The day following the behavior assessments, mice were then sacrificed for whole‐cell patch‐clamp recordings and further biochemical analyses. In this study, we demonstrated that microglia exhibit a senescent phenotype, as indicated by the presence of p16^+^IBA1^+^ cells. To reduce senescent microglia, we utilized a senolytic treatment named ABT‐737 for 3 days before 7‐day LPS exposure (Figure 5A). The body weight was shown in Figure 5B and the result indicated that ABT‐737 treatment and LPS exposures both resulted in significant weight loss in contrast to the Control + corn oil group.

The heat maps and track charts of the OFT, Y‐maze, and NOR tests were shown in Figure 5C–E, respectively. Among these groups, there was no difference in total distance and mean speed in the OFT (Figure 5F, *F*(2, 42) = 0.9039, *p* = 0.4127; Control + corn oil/LPS + corn oil: *p* = 0.5251; LPS + corn oil/LPS + ABT‐737: *p* = 0.9899; Figure 5G, *F*(2, 42) = 0.8824, *p* = 0.4213; Control + corn oil/LPS + corn oil: *p* = 0.5276; LPS + corn oil/LPS + ABT‐737: *p* = 0.9922). In the Y‐maze test, there was a significant reduction in total numbers into the arms and spontaneous alternation in the LPS + corn oil compared with the Control + corn oil group, and this effect was ameliorated by ABT‐737 treatment (Figure 5H, *F*(2, 42) = 5.602, *p* = 0.0070; Control + corn oil/LPS + corn oil: *p* = 0.0193; LPS + corn oil/LPS + ABT‐737: *p* = 0.0134; Figure 5I, *F*(2, 42) = 9.369, *p* = 0.0004; Control + corn oil/LPS + corn oil: *p* = 0.0005; LPS + corn oil/LPS + ABT‐737: *p* = 0.0061). Moreover, mice in the LPS + corn oil group had a lower recognition index compared with the Control + corn oil group, which was also alleviated by ABT‐737 treatment (Figure 5K, *F*(2, 33) = 12.52, *p* < 0.0001; Control + corn oil/LPS + corn oil: *p* < 0.0001; LPS + corn oil/LPS + ABT‐737: *p* = 0.0413). The results suggested that cellular senescence plays a key role in working and recognition memory impairments induced by LPS.

To characterize the pharmacodynamic profile of ABT‐737, we quantitatively assessed temporal expression of senescent markers (p16, p21) and pro‐inflammatory cytokines (IL‐1β, TNF‐α, IL‐6) in the hippocampus on Days 3, 7, and 14 following ABT‐737 administration (Figure S4A). There was no significant difference in the level of IL‐1β, TNF‐α, and IL‐6 among these timepoints (Figure S4B,C, *F*(3, 20) = 0.3988, *p* = 0.7554; Corn oil/3 days post ABT‐737, *p* = 0.9998, Corn oil/7 days post ABT‐737, *p* = 0.8676, Corn oil/14 days post ABT‐737, *p* = 0.9010; Figure S4D, *F*(3, 20) = 1.817, *p* = 0.1765; Corn oil/3 days post ABT‐737, *p* = 0.9601, Corn oil/7 days post ABT‐737, *p* = 0.6090, Corn oil/14 days post ABT‐737, *p* = 0.7328; Figure S4E, *F*(3, 20) = 1.794, *p* = 0.1808; Corn oil/3 days post ABT‐737, *p* = 0.2787, Corn oil/7 days post ABT‐737, *p* = 0.2790, Corn oil/14 days post ABT‐737, *p* = 0.2337). These results indicated that ABT‐737 had no significant effect on the baseline inflammatory status. In addition, p16 expression levels showed a significant decrease on Day 14 post‐ABT‐737 treatment compared with the Corn oil group. However, no significant change was observed on Days 3 or 7 post‐ABT‐737 treatment (Figure S4F,G, *F*(3, 20) = 11.83, *p* = 0.0001; Corn oil/3 days post ABT‐737, *p* = 0.8552, Corn oil/7 days post ABT‐737, *p* = 0.0848, Corn oil/14 days post ABT‐737, *p* = 0.0011). However, the level of p21 was not significantly altered, although there was a decreasing tendency on Days 7 and 14 post‐ABT‐737 treatment compared with the Corn oil group (Figure S4H, *F*(3, 20) = 1.174, *p* = 0.3445; Corn oil/3 days post ABT‐737, *p* > 0.9999, Corn oil/7 days post ABT‐737, *p* = 0.5832, Corn oil/14 days post ABT‐737, *p* = 0.5241). Furthermore, there was no obvious astrocytic or microglial activation in the hippocampal CA1 on Days 3, 7, and 14 after ABT‐737 treatment (Figure S4I,J, *F*(3, 12) = 0.4380, *p* = 0.7299; Corn oil/3 days post ABT‐737, *p* = 0.8338, Corn oil/7 days post ABT‐737, *p* = 0.7468, Corn oil/14 days post ABT‐737, *p* = 0.7919; Figure S4K, *F*(3, 12) = 0.1772, *p* = 0.9098; Corn oil/3 days post ABT‐737, *p* = 0.9148, Corn oil/7 days post ABT‐737, *p* > 0.9999, Corn oil/14 days post ABT‐737, *p* = 0.9994).

### 2.5 Senolytic Therapy Reduced the Accumulation of Senescent Microglia in the CA1

**FIGURE 6:**
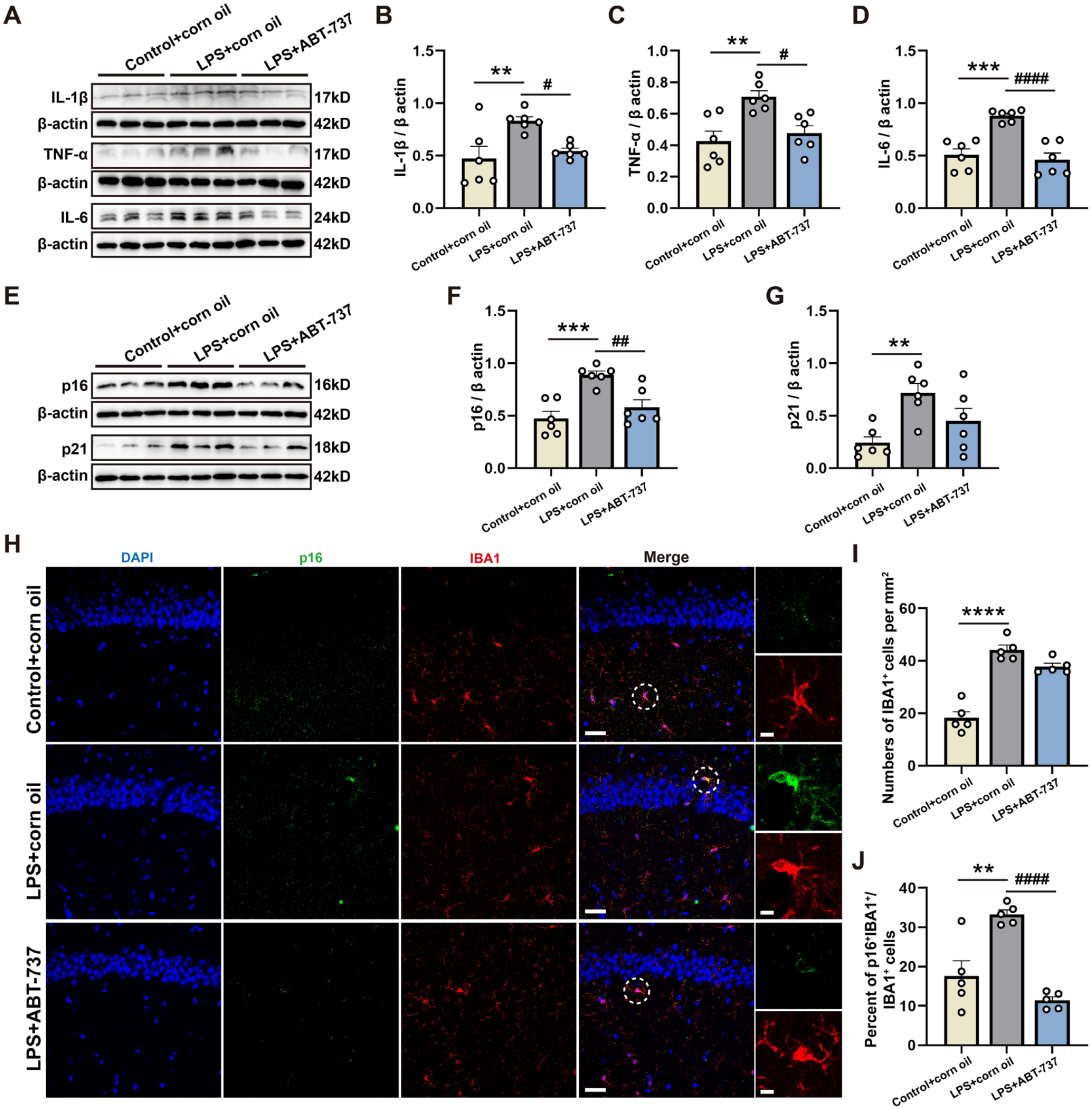
Senolytic therapy reduced the accumulation of senescent microglia in the CA1. (A) Quantification of normalized IL‐1β, TNF‐α and IL‐6 protein intensities. (B) Relative expression of IL‐1β/β actin in the hippocampi among experimental groups, *N* = 6 mice for each group. (C) Relative expression of TNF‐α/β actin in the hippocampi among experimental groups, *N* = 6 mice for each group. (D) Relative expression of IL‐6/β actin in the hippocampi among experimental groups, *N* = 6 mice for each group. (E) Quantification of normalized p16 and p21 protein intensities. (F) Relative expression of p16/β actin in the hippocampi among experimental groups, *N* = 6 mice for each group. (G) Relative expression of p21/β actin in the hippocampi among experimental groups, *N* = 6 mice for each group. (H) Representative images immunostained for DAPI^+^ (blue), p16^+^ (green) and IBA1^+^ (red) in the hippocampal CA1 among experimental groups. Scale bar = 20 μm, Zoom = 4 μm. (I) The number of p16^+^IBA1^+^ cells in the hippocampal CA1 among experimental groups, *N* = 5 mice for each group. (J) The percent of p16^+^IBA1^+^ cells in the hippocampal CA1 among experimental groups, *N* = 5 mice for each group. All values are shown as mean ± SEM by two‐tailed *t* test and by one‐way ANOVA with Tukey's post hoc test, **p < 0.01, ***p < 0.001, ****p < 0.0001, ^#^ *p* < 0.05, ^##^ *p* < 0.01, ^####^ *p* < 0.0001.

To verify whether LPS exposures result in microglial senescence, we measured the expressions of several SASPs, including TNF‐α, IL‐1β, and IL‐6, which were the key features of senescent cells (de Magalhaes 2024). The notable elevation of these SASPs was found in the hippocampi of mice in the LPS + corn oil group compared with the Control + corn oil group, which was relieved by ABT‐737 treatment (Figure 6A; *F*(2, 15) = 6.914, *p* = 0.0074; Control + corn oil/LPS + corn oil: *p* = 0.0094; LPS + corn oil/LPS + ABT‐737: *p* = 0.0386; Figure 6C, *F*(2, 15) = 8.828, *p* = 0.0029; Control + corn oil/LPS + corn oil: *p* = 0.0039; LPS + corn oil/LPS + ABT‐737: *p* = 0.0165; Figure 6D, *F*(2, 15) = 20.75, *p* < 0.0001; Control + corn oil/LPS + corn oil: *p* = 0.0003; LPS + corn oil/LPS + ABT‐737: *p* < 0.0001). Additionally, we measured the expressions of p16 and p21 in the hippocampi, and the results showed that LPS treatment induced significantly increased p16 and p21 levels in the hippocampi of mice. The increased expression of p16 was partly mitigated by ABT‐737 treatment (Figure 6F, *F*(2, 15) = 13.00, *p* = 0.0005; Control + corn oil/LPS + corn oil: *p* = 0.0006; LPS + corn oil/LPS + ABT‐737: *p* = 0.0069). However, ABT‐737 treatment did not reduce the increase of p21 level (Figure 6G, *F*(2, 15) = 6.547, *p* = 0.0090; Control + corn oil/LPS + corn oil: *p* = 0.0069; LPS + corn oil/LPS + ABT‐737: *p* = 0.1371). Accordingly, we concentrated on the p16 expression in microglia. By immunofluorescence, we found that LPS could induce the expression of p16 in microglia in the CA1 (Figure 6H), and the number of IBA1^+^ cells and the percent of p16^+^IBA1^+^ cells were significantly enhanced in the LPS + corn oil group compared with the Control + corn oil group (Figure 6I, *F*(2, 12) = 50.58, *p* < 0.0001; Control + corn oil/LPS + corn oil: *p* < 0.0001; LPS + corn oil/LPS + ABT‐737: *p* = 0.0868; Figure 6J, *F*(2, 12) = 22.07, *p* < 0.0001; Control + corn oil/LPS + corn oil: *p* = 0.0016; LPS + corn oil/LPS + ABT‐737: *p* < 0.0001). Although there was no change in the number of IBA1^+^ cells between the LPS + corn oil and LPS + ABT‐737 groups, the percent of p16^+^IBA1^+^ cells was reduced by ABT‐737 treatment in comparison to the LPS + corn oil group (Figure 6I,J).

Next, we examined microglia morphological features and found that senescent microglia exhibited distinct morphological characteristics in the LPS + corn oil group compared with that in the Control + corn oil group (Figure S5A,B). Specifically, there was a marked increase in the soma area and the number of endpoints per microglia, alongside a significant reduction in the maximum branch length, which were rescued by ABT‐737 (Figure S5C, *F*(2, 45) = 18.77, *p* < 0.0001; Control + corn oil/LPS + corn oil: *p* < 0.0001; LPS + corn oil/LPS + ABT‐737: *p* = 0.0160; Figure S5D, *F*(2, 45) = 32.99, *p* < 0.0001; Control + corn oil/LPS + corn oil: *p* < 0.0001; LPS + corn oil/LPS + ABT‐737: *p* < 0.0001; Figure S5E, *F*(2, 45) = 59.84, *p* < 0.0001; Control + corn oil/LPS + corn oil: *p* < 0.0001; LPS + corn oil/LPS + ABT‐737: *p* = 0.0094).

### 2.6 Senolytic Therapy Reduced Microglial Phagocytosis of Excitatory Synapses in the CA1

**FIGURE 7:**
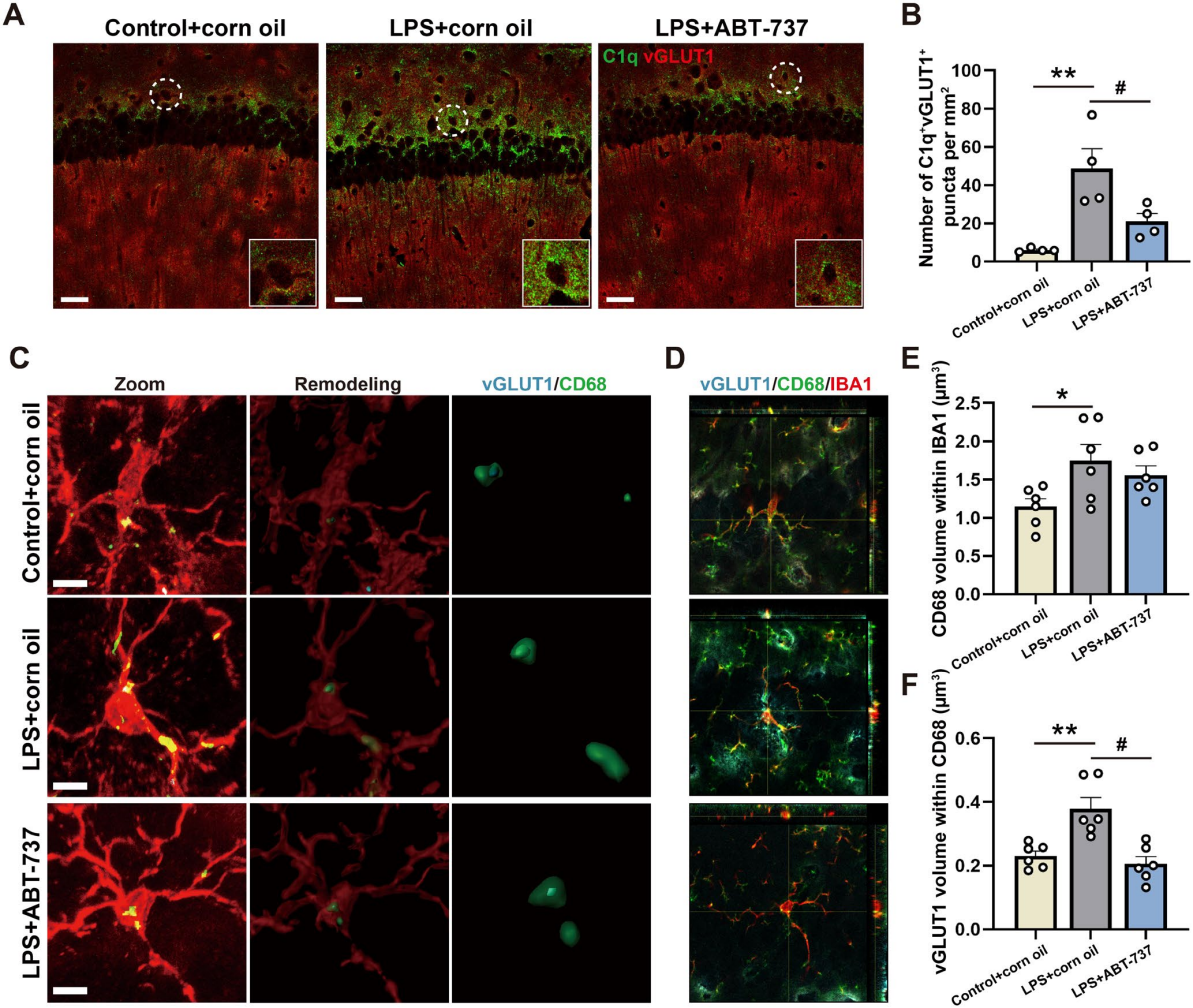
Senolytic therapy reduced microglial phagocytosis of excitatory synapses in the CA1. (A) Representative images immunostained for C1q^+^ (green) and vGLUT1^+^ (red) in the hippocampal CA1 among experimental groups. Scale bar = 20 μm. (B) The number of C1q^+^vGLUT1^+^ cells in the hippocampal CA1 among experimental groups, *N* = 4 mice for each group. (C) Representative images immunostained for vGLUT1^+^ (blue), CD68^+^ (green) and IBA1^+^ (red) in Zoom and for 3D reconstruction in the hippocampal CA1 among experimental groups, Scale bar = 4 μm. (D) Co‐localization of vGLUT1, CD68, and IBA1 puncta. (E) Quantification of CD68 volume within IBA1 volume in the hippocampal CA1 among experimental groups, *N* = 6 mice for each group. (F) Quantification of vGLUT1 volume within CD68 volume in the hippocampal CA1 among experimental groups, *N* = 6 mice for each group. All values are shown as mean ± SEM by one‐way ANOVA with Tukey's post hoc test, *p < 0.05, **p < 0.01, ^#^ *p* < 0.05.

As previously indicated, component C1q plays a key role in the process of synaptic pruning mediated by microglia. This complement protein had been demonstrated to tag excitatory synapses by phagocytotic signaling pathways of microglia (Li et al. 2024). We analyzed the expression of C1q and vGLUT1 in the CA1 (Figure 7A). It showed that the number of C1q^+^vGLUT1^+^ puncta was remarkably increased in the LPS + corn oil group compared with the Control + corn oil group, which was relieved in the LPS + ABT‐737 group (Figure 7B, *F*(2, 9) = 10.88, *p* = 0.0040; Control + corn oil/LPS + corn oil: *p* = 0.0033; LPS + corn oil/LPS + ABT‐737: *p* = 0.0376). Besides, the phagocytosis of excitatory synapses by microglia was also assessed (Figure 7C,D). Similarly, a significant enhancement in both the volume of microglial CD68 and the volume of vGLUT1 internalized within microglial CD68 was observed in the LPS + corn oil group compared with the Control + corn oil group (Figure 7E, *F*(2, 15) = 4.161, *p* = 0.0365; Control + corn oil/LPS + corn oil: *p* = 0.0327; LPS + corn oil/LPS + ABT‐737: *p* = 0.6613; Figure 7F, *F*(2, 15) = 13.12, *p* = 0.0005; Control + corn oil/LPS + corn oil: *p* = 0.0028; LPS + corn oil/LPS + ABT‐737: *p* = 0.0007). However, ABT‐737 treatment significantly attenuated the increased volume of vGLUT1 internalized within microglial CD68 (Figure 7F).

### 2.7 Senolytic Therapy Ameliorated LPS‐Induced Dendritic Spine Loss in the CA1

**FIGURE 8:**
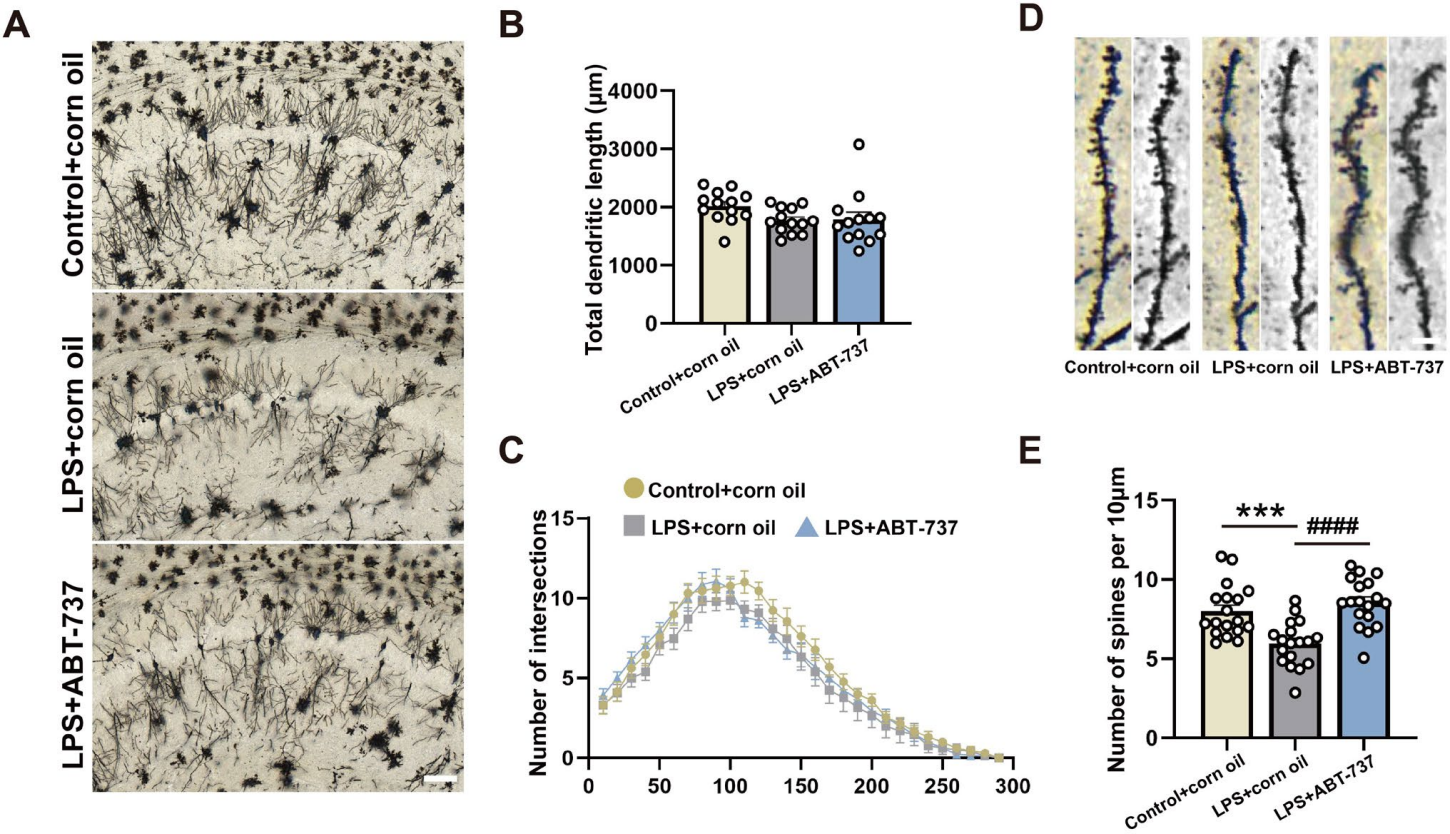
Senolytic therapy ameliorated LPS‐induced dendritic spine loss in the CA1. (A) Representative Golgi staining images of pyramidal neurons in the hippocampal CA1 among experimental groups. (B) Total dendritic length of pyramidal neurons in the hippocampal CA1 among experimental groups, *N* = 13 neurons from 3 mice for each group. (C) Number of intersections of pyramidal neurons in the hippocampal CA1 among experimental groups, *N* = 13 neurons from 3 mice for each group. (D) Representative Golgi staining images of dendritic spine density of hippocampal pyramidal neurons in the hippocampal CA1 among experimental groups. (E) Number of dendritic spines of hippocampal pyramidal neurons in the hippocampal CA1 among experimental groups, *N* = 18 dendrites from 3 mice for each group. All values are shown as mean ± SEM by one‐way ANOVA with Tukey's post hoc test, ***p < 0.001, ^####^ *p* < 0.0001.

To investigate the impact of LPS exposures and ABT‐737 therapy on dendritic spine in mice. We measured dendritic spine density in the hippocampal CA1 after LPS and ABT‐737 treatment (Figure 8A). Golgi staining showed that the total dendritic length and intersection number were not significantly altered among these groups (Figure 8B, *F*(2, 36) = 2.288, *p* = 0.1160; Control + corn oil/LPS + corn oil: *p* = 0.1475; LPS + corn oil/LPS + ABT‐737: *p* = 0.9886; Figure 8C). Nevertheless, the number of dendritic spines was remarkably decreased in the CA1 after LPS exposures, and this effect was rescued by ABT‐737 treatment (Figure 8D,E, *F*(2, 51) = 14.21, *p* < 0.0001; Control + corn oil/LPS + corn oil: *p* = 0.0006; LPS + corn oil/LPS + ABT‐737: *p* < 0.0001).

### 2.8 Senolytic Therapy Ameliorated LPS‐Induced Abnormal Miniature Excitatory Synaptic Currents (mEPSCs) and Long‐Term Potential (LTP) Impairment in the CA1

**FIGURE 9:**
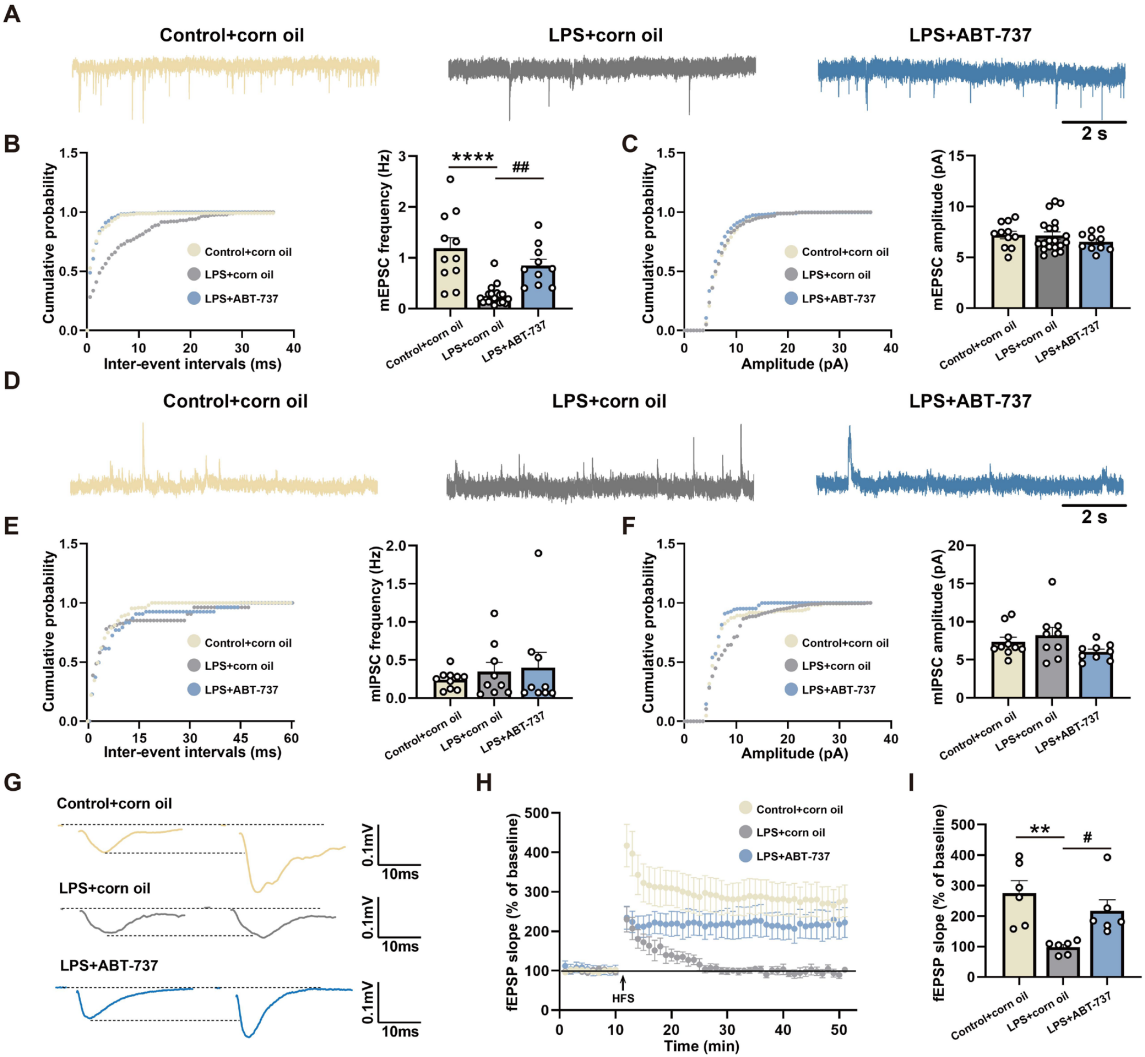
Senolytic therapy ameliorated LPS‐induced abnormal mEPSCs and LTP impairment in the CA1. (A) Representative sample traces of mEPSCs in the hippocampal CA1 among experimental groups. (B) Cumulative distribution and quantification of mEPSC frequency in the hippocampal CA1 among experimental groups, *N* = 10–20 neurons from 6 mice for each group. (C) Cumulative distribution and quantification of mEPSC amplitude in the hippocampal CA1 among experimental groups, *N* = 10–20 neurons from 6 mice for each group. (D) Representative sample traces of mIPSCs in the hippocampal CA1 among experimental groups. (E) Cumulative distribution and quantification of mIPSC frequency in the hippocampal CA1 among experimental groups, *N* = 9–10 neurons from 6 mice for each group. (F) Cumulative distribution and quantification of mIPSC amplitude in the hippocampal CA1 among experimental groups, *N* = 9–10 neurons from 6 mice for each group. (G) Representative traces of fEPSPs in the hippocampal CA1 among experimental groups. (H, I) Quantification of fEPSP slope in the hippocampal CA1 among experimental groups, *N* = 6 mice for each group. All values are shown as mean ± SEM by one‐way ANOVA with Tukey's post hoc test, **p < 0.01, ****p < 0.0001, ^#^ *p* < 0.05, ^##^ *p* < 0.01.

Next, we performed whole‐cell patch‐clamp recording to investigate the effect of LPS on the excitability of pyramidal neurons. The basic synaptic transmission of pyramidal neurons in the CA1 was determined with the amplitude and frequency of mEPSCs and miniature inhibitory synaptic currents (mIPSCs) (Figure 9A,D). The results showed that the mEPSC frequency was significantly reduced after LPS exposures, which was rescued by ABT‐737 treatment (Figure 9B, *F*(2, 38) = 18.05, *p* < 0.0001; Control + corn oil/LPS + corn oil: *p* < 0.0001; LPS + corn oil/LPS + ABT‐737: *p* = 0.0028). However, the mEPSC amplitude exhibited no significant change among groups (Figure 9C, *F*(2, 38) = 0.8030, *p* = 0.4555; Control + corn oil/LPS + corn oil: *p* = 0.9923; LPS + corn oil/LPS + ABT‐737: *p* = 0.4935). In addition, there was no alteration in the amplitude and frequency of mIPSCs among groups (Figure 9E, *F*(2, 25) = 0.3866 *p* = 0.6833; Control + corn oil/LPS + corn oil: *p* = 0.8327; LPS + corn oil/LPS + ABT‐737: *p* = 0.9602; Figure 9F, *F*(2, 25) = 2.162, *p* = 0.1362; Control + corn oil/LPS + corn oil: *p* = 0.6934; LPS + corn oil/LPS + ABT‐737: *p* = 0.1187). We next examined the impact of LPS exposures and ABT‐737 therapy on neuronal synaptic plasticity. LTP induction measured by the field excitatory postsynaptic potential (fEPSP) slope was significantly decreased in the LPS + corn oil group compared with the Control + corn oil group, while the administration of ABT‐737 significantly prevented this reduction (Figure 9G–I, *F*(2, 15) = 7.973, *p* = 0.0044; Control + corn oil/LPS + corn oil: *p* = 0.0037; LPS + corn oil/LPS + ABT‐737: *p* = 0.0468).

## 3 Discussion

The loss of excitatory synapses linked to cognitive dysfunction has been documented in various neurodegenerative diseases (Gomez‐Murcia et al. 2024; Li et al. 2024; Jia et al. 2025). In the current study, we uncovered a driving role for senescent microglia in excitatory synapse elimination and the aggravation of cognitive dysfunction in the mouse model of neuroinflammation. In contrast, senolytic ABT‐737 treatment prevented excitatory synapse loss and cognitive dysfunction, indicating that the clearance of senescent microglia could be a potential therapeutic target for alleviating cognitive dysfunction by neuroinflammation (Graphical abstract).

Neuroinflammation is defined as an inflammatory response occurring within the CNS, specifically in the brain or spinal cord (Chen et al. 2025). Cumulative evidence indicates that neuroinflammation is implicated in an increasing array of neuropsychiatric disorders (Pape et al. 2019; Han et al. 2021), particularly those linked to the decline in cognitive function. The administration of LPS has been extensively utilized as a model for studying neuroinflammation, providing substantial evidence regarding the adverse effects of inflammation on neurogenic processes (Dominguez‐Rivas et al. 2021). In order to induce a microenvironment characterized by neuroinflammation, we established a mouse model involving the intraperitoneal administration of LPS at a dosage of 0.5 mg/kg over a period of 7 days. This model has been well demonstrated to result in cognitive dysfunction in our previous study (Wu et al. 2023; Liu et al. 2025). The hippocampal CA1 region plays a pivotal role in regulating cognitive functions through its unique synaptic connectivity architecture and spatiotemporal coding properties (Qian et al. 2025; Uytiepo et al. 2025). Serving as the primary output node of the hippocampus, the functional integrity of the CA1 is crucial for the formation and retrieval of episodic memory (Ramsaran et al. 2025). A previous study demonstrated that the CA1 exhibited remarkable vulnerability to neurodegenerative changes, with progressive loss of pyramidal neurons occurring in early‐stage AD (Mostafa et al. 2025). By contrast, CA1 lesions impaired spatial and object recognition memory (Gonzalez et al. 2025). Thus, our present study focused on the hippocampal CA1 region.

Cellular senescence is defined by a permanent cessation of the cell cycle, accompanied by a flattened and enlarged cellular morphology, resistance to programmed cell death (apoptosis), and changes in gene expression, particularly involving p16, p21, and p53 (Lucas et al. 2023). Additionally, it is marked by modifications in chromatin structure, the expression of SA‐β‐gal, and the development of SASPs such as IL‐1β, TNF‐α, and IL‐6 (Lucas et al. 2023). An increasing amount of research suggests that age‐related DNA damage and cellular senescence in the immune system and CNS significantly contribute to the exacerbation of neuroinflammation and cognitive deterioration during typical brain aging (Zhang et al. 2024). Our results showed that neuroinflammation induced by LPS resulted in increased secretion of multiple SASPs, as well as up‐regulated p16 and p21, suggesting that cellular senescence may underlie neuroinflammation‐induced cognitive dysfunction.

The aggregation of senescent microglia, along with their altered immune functions and interactions with other brain cells, may significantly contribute to the pathogenesis of aging‐related neurodegenerative conditions (Rim et al. 2024). On the one hand, senescent microglia exhibit similar features to other senescent cells, including DNA damage, reactive oxygen species (ROS) generation, abnormal lipid droplets and protein aggregates, and SASPs secretion (Wang et al. 2025). In addition, senescent microglia appear to have a distinct signature compared with other senescent cells and other subtypes of microglia. For example, morphological variation characterized by cytoplasmic fragmentation and swelling is observed in the senescent microglia; however, it does not consistently correlate with other canonical markers of senescence (Malvaso et al. 2023). Additionally, ferritin represents the primary immunophenotypic alteration to distinguish senescent microglia from dystrophic microglia (Neumann et al. 2023). Our results suggested that there may exist senescent microglia with enhanced lysosomal function in the hippocampi of the neuroinflammation model by scRNA‐seq analysis. Besides, by immunofluorescence validation, we found a senescent subtype of microglia, characterized by highly expressed levels of p16, a classic hallmark of cellular senescence, was significantly present in the hippocampal CA1. Moreover, the senescent microglia highly expressing p16 exhibited markedly distinct morphological characteristics in comparison to normal microglia, including an enlarged cell body, a reduction in branch length, and a decreased number of endpoints. Interestingly, our findings indicated that the percentage of microglia with significant SA‐β‐gal activity did not exhibit a significant difference between the control and LPS groups. Therefore, we proposed that SA‐β‐gal activity cannot be regarded as a specific marker of senescent microglia, as it appears to be associated with elevated lysosomal quantity or activity rather than senescence itself (Lee et al. 2006; Piechota et al. 2016). We also examined two other brain subregions, namely the DG and the PrL, which are closely associated with working and recognition memory (Xu, Han, et al. 2023; Xu, Liu, et al. 2023; Scott et al. 2024). However, no similar microglial cellular response was observed in these regions. These results suggested that neuroinflammation‐induced senescent microglia are region dependent.

Synaptic plasticity plays a crucial role in the developing neural circuits, and the maintenance of its homeostasis is essential for supporting cognitive functions in neurodevelopmental and neuropsychiatric disorders (Connor and Siddiqui 2023). Moreover, plasticity deficits in neural pathways are often linked to behavioral indicators of impaired cognition in animal models of neurological disorders (Getz et al. 2022; Hashimoto et al. 2023). The precise removal of redundant synapses is crucial for the proper development and functional equilibrium of a healthy brain (Eyo and Molofsky 2023). This process necessitates the interplay of both positive and negative signals that regulate synaptic elimination. Substantial evidence has established that microglia are integral to the process of synaptic remodeling (Eyo and Molofsky 2023). Complement proteins, specifically C1q and C3, have been demonstrated to promote microglial engulfment of synaptic structures through the activation of the C3 receptor, thereby contributing to synapse loss in a mouse model of AD (Hong et al. 2016). Furthermore, the SIRPα‐CD47 signaling axis has been identified to negatively regulate microglial phagocytosis and synaptic pruning during early neurodevelopment (Lehrman et al. 2018; Ding et al. 2021). In this study, we observed that the phagocytic activity of synapses by microglia was significantly increased in the CA1 following LPS exposures, with a notable specificity for excitatory synapses, as opposed to inhibitory synapses. Meanwhile, the electrophysiology recording in vitro also validated that, which showed a reduced frequency of mEPSCs but no change in mIPSCs. Furthermore, a large amount of C1q was detected in conjunction with excitatory synaptic structures induced by LPS. These results indicated that microglia selectively engulf excitatory synapses in our model. Prior research indicated that microglia mediated synapse engulfment and the subsequent loss of excitatory synapses via the C1q pathway, which aligns with our findings (Xu, Han, et al. 2023; Xu, Liu, et al. 2023; Zhou et al. 2023). However, a recent study posited that complement C3 served as the primary molecular regulator that enhances microglial engulfment of excitatory synapses in a mouse model of fracture surgery (Li et al. 2024). Therefore, we speculated that the discrepancies between our study and others could be largely attributed to differences in the mouse models used.

It is worth mentioning that microglia exhibited increased phagocytosis of excitatory synapses in our study, as evidenced by elevated engulfment of synaptic markers vGLUT1. However, this heightened phagocytic activity may not translate to enhanced synaptic clearance, as lysosomal overload and incomplete degradation could disrupt functional consequences (Qin et al. 2023). In neurodegenerative contexts, lysosomal expansion often reflects cellular stress responses rather than improved clearance efficiency. For instance, microglia in AD models accumulate Aβ‐laden lysosomes with impaired acidification, despite up‐regulated lysosomal biogenesis, ultimately failing to resolve pathological aggregates (Zhang et al. 2022; Mancano et al. 2024). This apparent paradox of heightened synaptic uptake alongside lysosomal dysfunction underscores the vital difference between phagocytic capacity and lysosomal degradative efficiency (Quick et al. 2023).

Aging is recognized as a primary risk factor for most neurodegenerative disorders, where senescence contributes to the development of age‐related pathologies (Robbins et al. 2021; Kudlova et al. 2022). There is thus considerable interest in mitigating or eradicating the burden of cellular senescence, which has led to the emergence of senolytics (Kirkland and Tchkonia 2020). Senolytics are a category of drugs that selectively target and clear senescent cells. ABT‐737 is a well‐established senolytic drug that exerts effects by inhibiting Bcl‐2 family proteins associated with apoptosis (Oltersdorf et al. 2005). A recent study demonstrated that ABT‐737 specifically eliminated senescent microglia, resulting in enhanced cognitive function and reduced neuroinflammation (Rachmian et al. 2024). In this study, we utilized ABT‐737 to investigate the role of senescent microglia in the neuroinflammation mouse model. Our results showed that ABT‐737 improved cognitive dysfunction in mice following LPS exposures and reduced the accumulation of senescent microglia expressing p16. A prior investigation reported that highly p16‐expressing macrophage ablation by ABT‐737 decreased tumor burden by promoting immunosurveillance in a KRAS‐driven lung cancer model (Haston et al. 2023). Besides, ABT‐737 rescued the dendritic spine loss and improved the LTP impairment in the CA1. These results suggested that senescent microglia and their effect on synaptic plasticity may be involved in cognitive impairments in the neuroinflammation model.

Notably, our experimental design incorporated a 3‐day ABT‐737 preconditioning regimen initiated 7 days prior to LPS exposure and 14 days prior to subsequent behavioral assessment. This timepoint was empirically determined based on our data demonstrating that the peak geroprotective effects was on Day 14 post ABT‐737 treatment. Despite theoretical concerns that ABT‐737 might modulate baseline immunity prior to inflammatory challenge, the data revealed no statistically significant changes in either hippocampal cytokine profiles or CA1 glial activation. However, we acknowledge that this design did not assess the senolytic efficacy of ABT‐737 in established neuroinflammatory conditions. Thus, our results should be interpreted as providing mechanistic insight into the role of pre‐existing senescent cells in inflammation susceptibility, rather than as evidence of post‐inflammation therapeutic efficacy. While our current data support central effects of ABT‐737, its broad‐targeting properties prevent full exclusion of systemic influences. Our future studies will utilize CNS‐specific delivery approaches (e.g., intrathecal injection) to distinguish between central and peripheral mechanisms.

Several limitations should be acknowledged in our study. Firstly, gender difference exhibits differential immune response sensitivity to LPS (Cai et al. 2016; Dockman et al. 2022), the study thus focused on male mice to minimize experimental variability. Secondly, we did not fully distinguish senescent microglia from other subpopulations, a challenge widely recognized in the field due to the current lack of specific molecular markers (Antignano et al. 2023; Ng et al. 2023; Rim et al. 2024). Thirdly, while the mechanisms underlying microglial senescence‐induced cognitive dysfunction were not investigated in this study, evidence suggests that the cGAS‐STING pathway is a key driver of senescence‐associated neuroinflammation and neurodegeneration in AD models (Carling et al. 2024; Yuan et al. 2025), a mechanism that warrants investigation in our future studies.

## 4 Conclusion

In summary, our study indicates that selective phagocytosis of excitatory synapses by senescent microglia contributes to cognitive dysfunction induced by LPS. Future therapeutic strategies for neurodegenerative disorders related to neuroinflammation may benefit from targeting senescent cells, particularly senescent microglia.

## 5 Methods

### 5.1 Animals and Model of Neuroinflammation

All experiments were reviewed and approved by the Animal Core Facility of Nanjing Medical University (Jiangsu, China). C57BL/6J male mice aged 8–10 weeks old were originally purchased from GemPharmatech Co. Ltd. (Jiangsu, China). Mice were housed in a light/dark (12 h/12 h) cycle environment at room temperature (RT, 22°C ± 2°C, 30%–60% humidity) and had ad libitum access to standard irradiated pelleted chow. For each experiment, C57BL/6J male mice were randomly selected. All animal experiments followed the Ethics Committee of Nanjing Medical University, and all procedures were performed in accordance with the relevant guidelines and regulations.

For establishing a model of neuroinflammation, mice underwent intraperitoneal injections of LPS (L2880‐100MG, Sigma‐Aldrich, 0.5 mg/kg, 0.1 mL) dissolved in normal saline. In contrast, the control group received an equivalent volume of normal saline. The LPS was administered daily at 9 am for consecutive 7 days, with a 24‐h interval maintained between each dose.

### 5.2 Senolytic Treatment

To achieve the removal of senescent cells, mice received 3 consecutive daily intraperitoneal injections of ABT‐737 (S1002‐50MG, Selleck Chemicals, 25 mg/kg; 0.3 mL) or a vehicle control. The ABT‐737 and the DMSO‐based vehicle were formulated into corn oil (8001‐30‐7, MedChemExpress). The ABT‐737 treatment was conducted daily at 9 am over a period of 3 days, with a 24‐h interval maintained between each dose, implemented 2 weeks prior to the behavioral assessment (Figure 5A) (Reyes et al. 2022).

### 5.3 Behavioral Tests

Behavioral assessments were conducted 24 h following the final LPS administration (Liu et al. 2025). The experimental protocol included OFT and NOR habituation on the first day, followed by Y‐maze and NOR training on the second day, and concluding with NOR testing on the third day.

#### 5.3.1 OFT

Locomotor activity was assessed in the OFT by individually positioning mice in the central region of a PVC chamber (50 cm × 50 cm × 30 cm). Their unrestricted movement was recorded for 5 min utilizing an overhead camera in conjunction with tracking software ANY‐maze (Stoelting, America).

#### 5.3.2 Y‐Maze Test

In the spontaneous alternation test, mice were positioned at the center of a Y‐shaped maze consisting of three arms labeled respectively as A, B, and C, each measuring 35 cm, and were permitted to navigate the maze freely for 8 min. The total number of entries into each arm, as well as the sequence of these entries, was meticulously recorded utilizing an overhead camera in conjunction with tracking software ANY‐maze (Stoelting, America). The percentage of spontaneous alternation was calculated as the ratio of successive entries into each of the three arms on overlapping triplet sets (actual alternations, such as ABC, BCA, and CBA) to total alternations (total arm entries −2) multiplied by 100. Additionally, the total number of entries was recorded as an indicator of locomotor activity. An arm entry was recorded when all four limbs of the mice were within the confines of the arm. The short‐term spatial working memory was quantified using the spontaneous alternation.

#### 5.3.3 NOR

The NOR test was performed utilizing an open field apparatus. The experimental procedure comprised three distinct sessions: habituation, training, and testing, each separated by a 24‐h interval. In the habituation session, mice were introduced to the open field apparatus and permitted to explore for 10 min. During the training session, two identical and familiar objects, referred to as F1 and F2, were positioned 20 cm from the walls of the apparatus, and the mice were again allowed to explore for 10 min. In the subsequent testing session, the mice were placed back in the apparatus, where one of the familiar objects was substituted with a novel object, referred to as N1, which differed in color and shape. The mice were allowed to explore for an additional 10 min. The position of N1 was randomized between each mouse and each group tested. The total exploration time with each object was meticulously recorded utilizing an overhead camera in conjunction with tracking software ANY‐maze (Stoelting, America). Object exploration was deemed valid only if the total exploration time for each mouse exceeded 5 s. Notably, the mice exhibited no object preference during the training session (Figure 5J). The recognition memory was quantified using a discrimination index, calculated as the ratio of time spent exploring N1 to the total time spent exploring both objects.

### 5.4 scRNA‐Seq and Analysis

#### 5.4.1 Single Cell Dissociation

The mice were euthanized at 8–10 weeks of age, and the hippocampal tissues from two mice in the control and LPS groups were extracted, respectively. Single‐cell dissociation was performed on ice under sterile conditions. Prior to the procedure, mice were anesthetized with 1.25% tribromoethanol and subsequently subjected to transcardial perfusion with 50 mL ice‐cold Hank's Balanced Salt Solution (HBSS, 21‐022‐CM, Corning). Following this, the brains were carefully dissected, and the hippocampi were isolated. The isolated hippocampi were minced with a razor blade and incubated in 10 mL of accutase (A1110501, Thermo Fisher) for 30 min at 4°C. The resulting cell suspension was then centrifuged at 300 g for 10 min at 4°C. After decanting the accutase, the cell pellet was resuspended in HBSS, and the resuspended cells were filtered through a 70‐μm cell strainer. We employed the 10× Genomics Chromium Single‐Cell 3′ Library & Gel Bead Kit v3 (10× Genomics, America), following the manufacturer's protocols. Specifically, we combined a suspension of single cells with the reverse transcription polymerase chain reaction (RT‐PCR) master mix, gel beads, and partitioning oil, subsequently loading this mixture into a Single‐Cell A Chip designed for the 10× Genomics system.

#### 5.4.2 Single Cell Library Preparation

Single‐cell library preparation was performed by the GENE DENOVO (Guangzhou, China). A concentration of 400,000 cells per milliliter was necessary to achieve a targeted recovery of approximately 10,000 cells per sample. The cell suspension, master mix, thawed Gel Beads, and partitioning oil were introduced into a Chromium Single Cell G chip. Subsequently, the filled chip was placed into the Chromium Controller, where each sample underwent processing, resulting in the partitioning of individual cells into uniquely labeled Gel Beads‐In‐Emulsion (GEMs). The GEMs were then extracted from the chip and subjected to reverse transcription, GEM dissolution, and cDNA purification at the laboratory bench. The resultant cDNA comprised a collection of uniquely barcoded molecules. A fraction of the purified and quantified pooled cDNA proceeded to library construction, during which standard Illumina sequencing primers and a unique i7 Sample index from 10× Genomics were incorporated into each cDNA pool. All cDNA pools and the resulting libraries were quantified using Qubit High Sensitivity assays (Thermo Fisher, America) and Agilent Bioanalyzer High Sensitivity chips (Agilent, America). The libraries were sequenced to yield between 40,000 and 50,000 fragment reads per cell, adhering to Illumina's standard protocol with the Illumina cBot and HiSeq 3000/4000 PE Cluster Kit. Sequencing of the flow cells was conducted as 150 × 2 paired‐end reads on an Illumina HiSeq 4000 HD, utilizing the HiSeq 3000/4000 sequencing kit and HCS v3.4.0.38 collection software. Base‐calling was executed using software Real‐Time Analysis.

#### 5.4.3 scRNA‐Seq Analysis

We employed Omicsmart Share (Guangzhou, China) for the alignment, filtering, barcode counting, and unique molecular identifier (UMI) counting of the single‐cell FASTQ files, followed by cluster analysis. The data from the four replicates were aggregated, and cells were subjected to filtration criteria, ensuring that only those exhibiting between 500 and 4000 expressed genes, between 1000 and 8000 UMIs, and a mitochondrial gene content of less than 10% of the total UMI were retained. Additionally, cells with a UMI count exceeding the mean UMI of the entire dataset plus three times the standard deviation were excluded from the analysis. Following stringent quality control filtering, a total of 42,069 high‐confidence cells were retained for downstream analyses, comprising 18,683 cells from two control biological replicates and 23,386 cells from two LPS‐treated biological replicates (hippocampi pooled from 3 mice per replicate). Subsequently, the data was subjected to Principal Component Analysis (PCA) to reduce its dimensionality to the top 31 principal components. Following this, 7 distinct cell clusters, including glutaminergic neurons, GABAergic neurons, oligodendrocytes, microglia, neuroblasts, oligodendrocyte progenitor cells, and astrocytes, were delineated utilizing clustering algorithms, and these clusters were visualized through a t‐distributed Stochastic Neighbor Embedding (t‐SNE) plot based on the aforementioned principal components. An analysis of microglia was then conducted to assess gene differences between the control group and the LPS group, which led to the identification of 577 highly variable genes, comprising 241 up‐regulated genes and 336 down‐regulated genes. Furthermore, a Kyoto Encyclopedia of Genes and Genomes (KEGG) enrichment analysis was performed on the differentially expressed genes (DEGs) identified between the microglia and other clusters. A modularity optimization procedure utilizing the Louvain algorithm was employed to iteratively aggregate cells, aiming to enhance the standard modularity function. A resolution parameter of 0.5 was selected to regulate the granularity of the resulting clusters. The identification of cluster marker genes was conducted using the SingleR function for each cluster, with subsequent filtering based on a Bonferroni‐corrected *p*‐value threshold of less than 0.05, a log2 fold change exceeding 0.36, and expression in a minimum of 10% of the cells within the cluster.

### 5.5 Immunofluorescence Staining

#### 5.5.1 Tissue Preparation

Mice were subjected to acute and deep anesthesia prior to undergoing transcardial perfusion with 50 mL of 1× PBS, followed by the administration of ice‐cold 4% paraformaldehyde. Following this procedure, the brains were extracted and further fixed in 4% PFA at 4°C for over 12 h. Subsequently, the brains were transferred to solutions of 15% and 30% sucrose in 1× PBS for cryoprotection. The brains were then processed into coronal slices with a thickness of 30 μm using a vibratome (Model VT1000S, Leica, Germany).

#### 5.5.2 Immunostaining of Brain Sections

The slices were briefly washed with 1× PBS for about 20 min, followed by incubation in 2% Triton X‐100 in 1× PBS for 30 min and 10% goat serum in 1× PBS for 2 h at RT. The slices were incubated with primary antibodies overnight at 4°C and with secondary antibodies for 2 h at RT. The primary antibodies used were: rabbit anti‐IBA1 (1:1000, 019‐19741, WAKO); mouse anti‐IBA1 (1:500, MABN92‐25UG, Sigma‐Aldrich); rat anti‐CD68 (1:500, AB53444, Abcam); rabbit anti‐beta Galactosidase (β‐Gal, 1:500, A11132, Thermo Fisher); mouse anti‐p16 (1:500, MA5‐17142, Thermo Fisher); mouse anti‐p21 (1:500, 556430, BD Biosciences); rat anti‐C1q (1:500, ab11861, Abcam); guinea pig anti‐vGLUT1 (1:500, 135304, Synaptic Systems); rabbit anti‐vGLUT1 (1:500, ab302688, Abcam); mouse anti‐vGAT (1:500, sc‐393373, Santa Cruz Biotechnology); rabbit anti‐GFAP (1:500, ab68428, Abcam); rabbit anti‐NEUN (1:500, 26975‐1‐AP, Proteintech). The secondary antibodies used were as follows: Alexa Fluor 594 goat anti‐rabbit IgG (1:400, 111‐585‐003, Jackson ImmunoResearch); Alexa Fluor 488 goat anti‐mouse IgG (1:400, 115‐545‐003, Jackson ImmunoResearch); FITC‐conjugated goat anti‐rat IgG (1:100, SA00003‐11, Proteintech); DyLight 405 goat anti‐mouse IgG (1:200, 33204ES60, YEASEN); DyLight 405 goat anti‐guinea pig IgG (1:200, 106‐475‐003, Jackson ImmunoResearch). In the final stage, the sections were mounted onto glass slides using an anti‐fade reagent and subsequently prepared for imaging with a laser confocal microscope (FV3000, Olympus, Japan).

#### 5.5.3 Quantitative Analysis

For quantitative analysis, three evenly spaced slices per mice were selected at 30 μm intervals spanning the rostrocaudal axis of the hippocampus, ensuring bilateral hemispheric inclusion through systematic random sampling. For the quantification of fluorescence intensities and numbers, images were analyzed by software ImageJ (NIH, America). Besides, the quantification of co‐localized synaptic puncta was performed using the ImageJ Puncta Analyzer plugin (NIH, America). For the quantification of synapse engulfment in microglia, images were acquired with a 40× oil objective with 3× digital zoom. Z‐stacks of 10 mm thickness from the middle of the slices were obtained with a 0.5 mm step size (Wen et al. 2023). Three microglia from each slice were captured for further analyses. Images then were processed and analyzed by software Imaris (Oxford Instruments, UK). The volumes of CD68 and IBA1 were quantified utilizing three‐dimensional surface rendering of confocal stacks in respective channels. For the assessment of vGLUT1 and vGAT engulfment by microglia, only corresponding puncta located within microglial CD68 structures were taken into account (Dundee et al. 2023; Wen et al. 2023). Furthermore, the morphological analyses of microglia were as described by the methodology outlined by Young and Morrison (Young and Morrison 2018). The acquired images were processed using ImageJ in conjunction with the AnalyzeSkeleton plugin (NIH, America). Briefly, the images were converted to binary format, followed by refinement procedures to eliminate extraneous components. Subsequently, the skeletonized images were subjected to analysis to evaluate the morphological characteristics of the microglia (Soni et al. 2024).

### 5.6 Western Blotting Analysis

Mice were subjected to deep anesthesia with 1.25% tribromoethanol, after which their brains were meticulously excised on ice. The hippocampi were isolated from the brain and subsequently stored at −80°C. Each hippocampus was lysed in an equal volume of 0.6 mL RIPA buffer supplemented with protease inhibitors (P0013B, Beyotime Biotechnology), 12 μL of phosphatase inhibitors (P1081, Beyotime Biotechnology), and 0.6 μL of protease inhibitor cocktails (KGP603, Keygentec) for 20 min on ice. The resulting lysates were centrifuged at 19,064 g for 15 min at 4°C to remove debris. Protein concentration was assessed using a BCA assay kit (P0010, Beyotime Biotechnology). Subsequently, equal quantities of protein samples were separated via either 12% or 15% SDS‐PAGE gels and transferred to a 0.45 μm thickness PVDF membrane (HVLP02500, Millipore). The separated proteins were then blocked with 3% skimmed milk for 1 h at RT, followed by overnight incubation with primary antibodies at 4°C. The primary antibodies included rabbit anti‐p16 (1:1000, AF1672, Beyotime Biotechnology), rabbit anti‐p21 (1:1000, AF5252, Beyotime Biotechnology), rabbit anti‐IL‐1β (1:1000, A16288, ABclonal), mouse anti‐TNF α (1:1000, 60291‐1‐Ig, Proteintech), rabbit anti‐IL‐6 (1:1000, A0286, ABclonal), and mouse anti‐β‐actin (1:10000, AB8226, Abcam). Following this, the membrane was incubated with secondary antibodies for 2 h at RT, including anti‐mouse HRP‐conjugated secondary antibody (1:10,000, SA00001‐2, Proteintech) and anti‐rabbit HRP‐conjugated secondary antibody (1:10,000, SA00001‐1, Proteintech). Immunoreactive bands were visualized using enhanced chemiluminescence with a gel documentation system (Bio‐Rad, America). The intensity of the protein bands was quantified using ImageJ software (NIH, America).

### 5.7 Golgi Staining

The FD Rapid Golgi Stain Kit (FD NeuroTechnologies, America) facilitated the morphological examination of neuronal dendrites and dendritic spines. Mice were anesthetized with 1.25% tribromoethanol, and their brains were harvested as quickly as possible. Fresh brains then were subjected to incubation with equal volumes of solutions A and B for 2 weeks at RT, within 5 mL Eppendorf tubes covered with aluminum foil to ensure darkness, respectively. Following an initial 24‐h incubation period, the impregnated solution was replaced. Subsequently, the tissues were transferred to solution C, which was also replaced after 24 h, and incubated at RT for an additional 3 days in the dark. The tissues were then sectioned into 150 μm slices using a vibratome (Model VT1000S, Leica, Germany) at −22°C, and stained in accordance with the manufacturer's instructions. Images were captured at 20× and 100× using an oil‐immersion objective (Olympus, Japan). The quantification of dendritic lengths, branching patterns, and spine density was performed utilizing the Neuro J and Sholl Analysis plugins available in ImageJ software (NIH, America).

### 5.8 Whole‐Cell Patch‐Clamp

#### 5.8.1 Acute Hippocampal Slice Preparation

Following the euthanasia of the mice, the whole brains were promptly excised and immersed in ice‐cold artificial cerebrospinal fluid (ACSF) composed of the following constituents: 185 mM sucrose, 20 mM D‐glucose, 26 mM NaHCO_3_, 2.5 mM KCl, 1.25 mM NaH_2_PO_4_, 1 mM CaCl_2_, and 6 mM MgCl_2_, which was saturated with a gas mixture of 95% O_2_ and 5% CO_2_, maintaining a pH of 7.4. Subsequently, hippocampal slices measuring 350 μm in thickness were prepared using a vibratome (Model VT1000S, Leica, Germany) and were subsequently placed in ACSF containing 124 mM NaCl, 20 mM D‐glucose, 26 mM NaHCO_3_, 2.5 mM KCl, 1.25 mM NaH_2_PO_4_, 1 mM CaCl_2_, and 6 mM MgCl_2_, also saturated with 95% O_2_ and 5% CO_2_, at a pH of 7.4. These slices were incubated for 30 min at 34°C and allowed to recover for 1 h at RT.

#### 5.8.2 mEPSCs and mIPSCs Recordings and Analysis

The slices were immersed in a recording chamber that maintained a continuous perfusion of 2 mL/min at RT using ACSF. Whole‐cell recordings were conducted on pyramidal neurons in the CA1, utilizing infrared optics with an upright microscope fitted with a 40× water‐immersion lens (BX51W1, Olympus, Japan) and an infrared‐sensitive CCD camera. The patch pipette, which exhibited an input resistance ranging from 3 to 6 MΩ, was filled with a solution comprising 125 mM D‐gluconate, 8 mM NaCl, 0.2 mM EGTA, 10 mM HEPES, 2 mM Mg‐ATP, and 0.3 mM NaGTP. The series resistances typically measured between 15 and 30 MΩ at the point of break‐in, with a compensation of 70% applied. Only pyramidal neurons demonstrating stable series resistance (with no more than 20% variation throughout the recording) were included in the analysis. Data acquisition was performed using an Axon patch 700B amplifier, with low‐pass filtering set at 2 kHz and digital sampling at 10 kHz, followed by analysis using software Clampfit (Molecular Devices, America). For mEPSCs and mIPSCs recordings, 1 μM tetrodotoxin and 10 μM bicuculline were incorporated into the bath solution of the pipettes. The analysis of mEPSCs and mIPSCs were conducted utilizing the Mini Analysis Program (Synaptosoft, America). The threshold for event detection for synaptic currents was set at 5 pA. Subsequently, a manual review of all detected events was conducted.

#### 5.8.3 LTP Assessment

The slices were afterward changed to a recording chamber that had a glass bottom filled with ACSF at 2 mL/min at RT. For recording the fEPSPs of the CA1 neurons, we put an electrode in the Schaffer collateral pathway to serve as the stimulating electrode. A stimulation intensity was selected that produced fEPSPs between 30% and 40% of the maximum response observed on the stimulus–response curve. LTP was induced through theta burst stimulation, which consisted of six episodes separated by 10‐s intervals. Each episode comprised five bursts at 5 Hz, with each burst consisting of five pulses delivered at 100 Hz. Following the tetanic stimulation, the field potential was recorded for 50 min. The extent of LTP was quantified as the percentage change in the average fEPSPs measured between 40 and 50 min after the initiation of LTP induction.

### 5.9 Statistical Analysis

Statistical analyses were conducted using software GraphPad Prism (GraphPad Software, USA). The results were expressed as mean ± standard error of the mean (SEM). Differences between two groups were examined using a two‐tailed *t* test, while differences among multiple means were assessed through a one‐way analysis of variance (ANOVA), followed by a Tukey's post hoc test as deemed appropriate. A significance level of *p* < 0.05 was established for determining statistically significant differences.

## Author Contributions

M.‐h.J. and J.‐j.Y. contributed to the conceptualization and supervision of the research; M.‐h.J. contributed to funding acquisition; K.L. wrote the manuscript; K.L. and D.F. analyzed the data; D.F., H.‐p.W., and X.‐y.H. revised the manuscript; and C.‐n.S., X.‐m.W., and Q.‐l.H. contributed to software and methodology; M.‐h.J. contributed to the review and editing of the manuscript. All authors read and approved the final manuscript.

## Conflicts of Interest

The authors declare no conflicts of interest.

## Supporting information


**Figure S1.** The level of p21 in the LPS‐induced microglia was not altered in the CA1. (A) Representative images immunostained for DAPI^+^ (blue), p21^+^ (green) and IBA1^+^ (red) in the hippocampal CA1 between the control and LPS groups. Scale bar = 20 μm, Zoom = 4 μm. (B) The number of p21^+^IBA1^+^ cells in the hippocampal CA1 between the control and LPS groups, *N* = 4 mice for each group. (C) The percent of p21^+^IBA1^+^ cells in the hippocampal CA1 between the control and LPS groups, *N* = 4 mice for each group. All values are shown as mean ± SEM by two‐tailed *t* test.

**Figure S2.** LPS did not alter the percent of senescent neurons or astrocytes in the CA1. (A) Representative images immunostained for DAPI^+^ (blue), p16^+^ (green) and GFAP^+^ (red) in the hippocampal CA1 between the control and LPS groups. Scale bar = 20 μm, Zoom = 4 μm. (B) The number of p16^+^GFAP^+^ cells in the hippocampal CA1 between the control and LPS groups, *N* = 4 mice for each group. (C) The percent of p16^+^GFAP^+^ cells in the hippocampal CA1 between the control and LPS groups, *N* = 4 mice for each group. (D) Representative images immunostained for DAPI^+^ (blue), p16^+^ (green) and NEUN^+^ (red) in the hippocampal CA1 between the control and LPS groups. Scale bar = 20 μm, Zoom = 4 μm. (E) The number of p16^+^NEUN^+^ cells in the hippocampal CA1 between the control and LPS groups, *N* = 4 mice for each group. (F) The percent of p16^+^NEUN^+^ cells in the hippocampal CA1 between the control and LPS groups, *N* = 4 mice for each group. All values are shown as mean ± SEM by two‐tailed *t* test, *p < 0.05.

**Figure S3.** LPS induced neuroinflammation but didn’t result in the accumulation of senescent microglia in the DG and the PrL. (A) Representative images immunostained for IBA1^+^ (green) and GFAP^+^ (red) in the PrL between the control and LPS groups. Scale bar = 20 μm. (B) The number of IBA1^+^ cells in the PrL between the control and LPS groups, *N* = 4 mice for each group. (C) The number of GFAP^+^ cells in the PrL between the control and LPS groups, *N* = 4 mice for each group. (D) Representative images immunostained for p16^+^ (green) and IBA1^+^ (red) in the PrL between the control and LPS groups. (E) Representative images immunostained for DAPI^+^ (blue), p16^+^ (green) and IBA1^+^ (red) in the PrL between the control and LPS groups. Scale bar = 20 μm, Zoom = 4 μm. (F) Representative images immunostained for IBA1^+^ (green) and GFAP^+^ (red) in the hippocampal DG between the control and LPS groups. Scale bar = 20 μm. (G) The number of IBA1^+^ cells in the hippocampal DG between the control and LPS groups, *N* = 4 mice for each group. (H) The number of GFAP^+^ cells in the hippocampal DG between the control and LPS groups, *N* = 4 mice for each group. All values are shown as mean ± SEM by two‐tailed *t* test, **p < 0.01, ***p < 0.001, ****p < 0.0001.

**Figure S4.** ABT‐737 had no remarkable effect on baseline inflammatory status. (A) Schematic representation of the experimental procedures. (B) Quantification of normalized IL‐1β, TNF‐α and IL‐6 protein intensities. (C) Relative expression of IL‐1β/β actin in the hippocampi among experimental groups, *N* = 6 mice for each group. (D) Relative expression of TNF‐α/β actin in the hippocampi among experimental groups, *N* = 6 mice for each group. (E) Relative expression of IL‐6/β actin in the hippocampi among experimental groups, *N* = 6 mice for each group. (F) Quantification of normalized p16 and p21 protein intensities. (G) Relative expression of p16/β actin in the hippocampi among experimental groups, *N* = 6 mice for each group. (H) Relative expression of p21/β actin in the hippocampi among experimental groups, *N* = 6 mice for each group. (I) Representative images immunostained for IBA1^+^ (green) and GFAP^+^ (red) in the hippocampal CA1 among experimental groups. Scale bar = 20 μm. (J) The number of IBA1^+^ cells in the hippocampal CA1 among experimental groups, *N* = 4 mice for each group. (K) The number of GFAP^+^ cells in the hippocampal CA1 among experimental groups, *N* = 4 mice for each group. All values are shown as mean ± SEM by one‐way ANOVA with Tukey’s post hoc test, **p < 0.01.

**Figure S5.** The morphological analysis of senescent microglia in the CA1. (A) Representative images immunostained for DAPI^+^ (blue), p16^+^ (green) and IBA1^+^ (red) in the hippocampal CA1 among experimental groups. Scale bar = 20 μm, Zoom = 8 μm. (B) Representative binary, outlined, and skeleton images for each microglia in the hippocampal CA1 among experimental groups. (C) Relative area of soma per microglia among experimental groups, *N* = 16 microglia from 4 mice for each group. (D) Maximum branch length per microglia among experimental groups, *N* = 16 microglia from 4 mice for each group. (E) The number of endpoints per microglia among experimental groups, *N* = 16 microglia from 4 mice for each group. All values are shown as mean ± SEM by one‐way ANOVA with Tukey’s.

## Data Availability

The data that support the findings of this study are available on request from the corresponding author. The data are not publicly available due to privacy or ethical restrictions.
