# Supplementary figures and images for: Senescent Microglia Mediate Neuroinflammation‐Induced Cognitive Dysfunction by Selective Elimination of Excitatory Synapses in the Hippocampal CA1
Source: Aging Cell. 2025 Jul 7;24(9):e70167. doi: 10.1111/acel.70167 (PMC12419858; doi:10.1111/acel.70167)

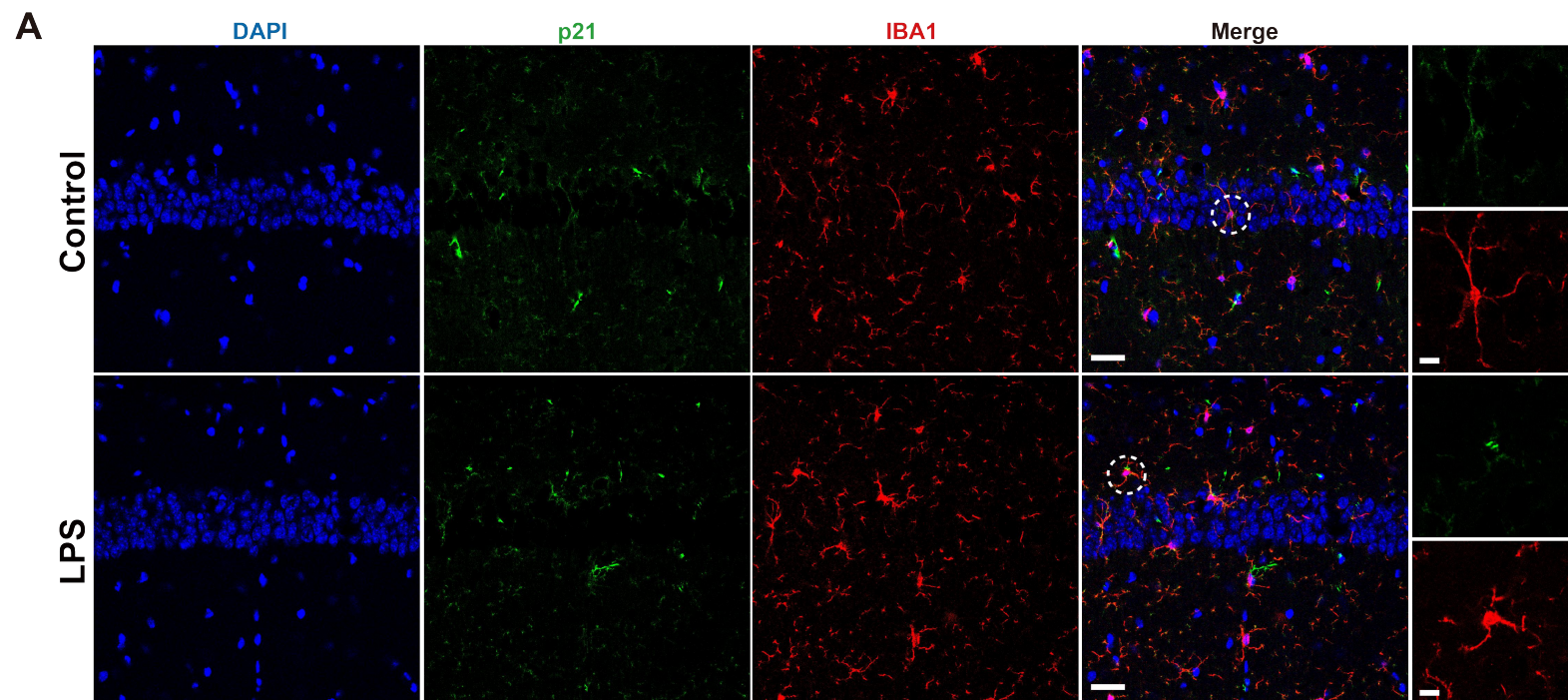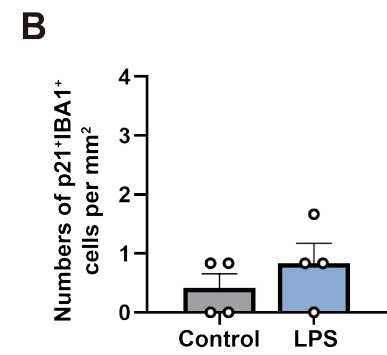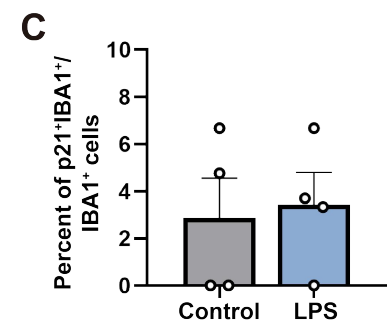

Supplement: Supplementary file 1 — Figure S1. The level of p21 in the LPS‐induced microglia was not altered in the CA1. (A) Representative images immunostained for DAPI+ (blue), p21+ (green) and IBA1+ (red) in the hippocampal CA1 between the control and LPS groups. Scale bar = 20 μm, Zoom = 4 μm. (B) The number of p21+IBA1+ cells in the hippocampal CA1 between the control and LPS groups, N = 4 mice for each group. (C) The percent of p21+IBA1+ cells in the hippocampal CA1 between the control and LPS groups, N = 4 mice for each group. All values are shown as mean ± SEM by two‐tailed t test. [file ACEL-24-e70167-s003.pdf]

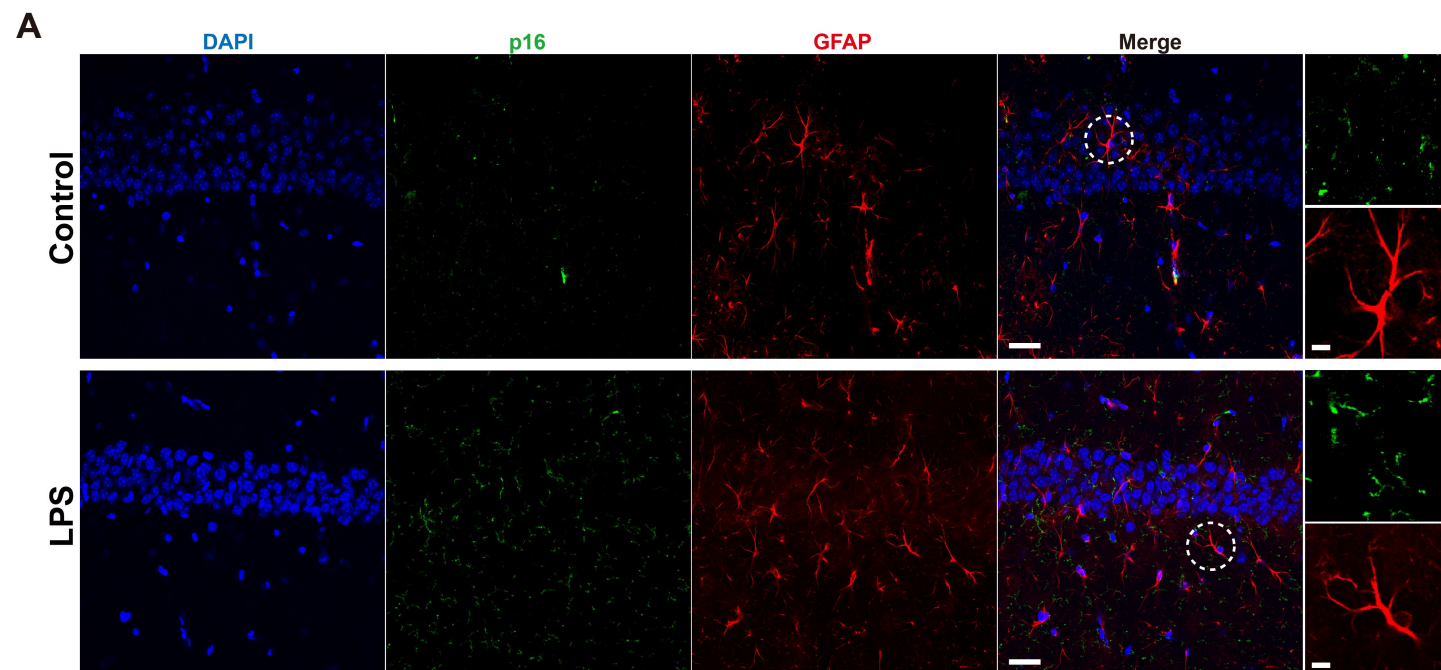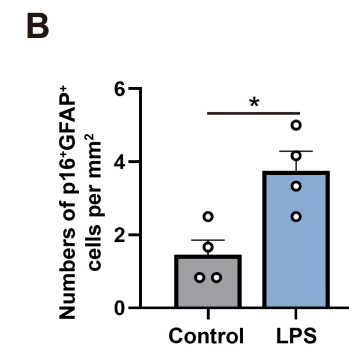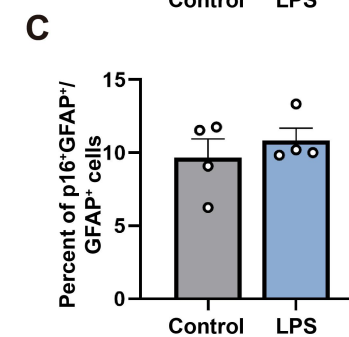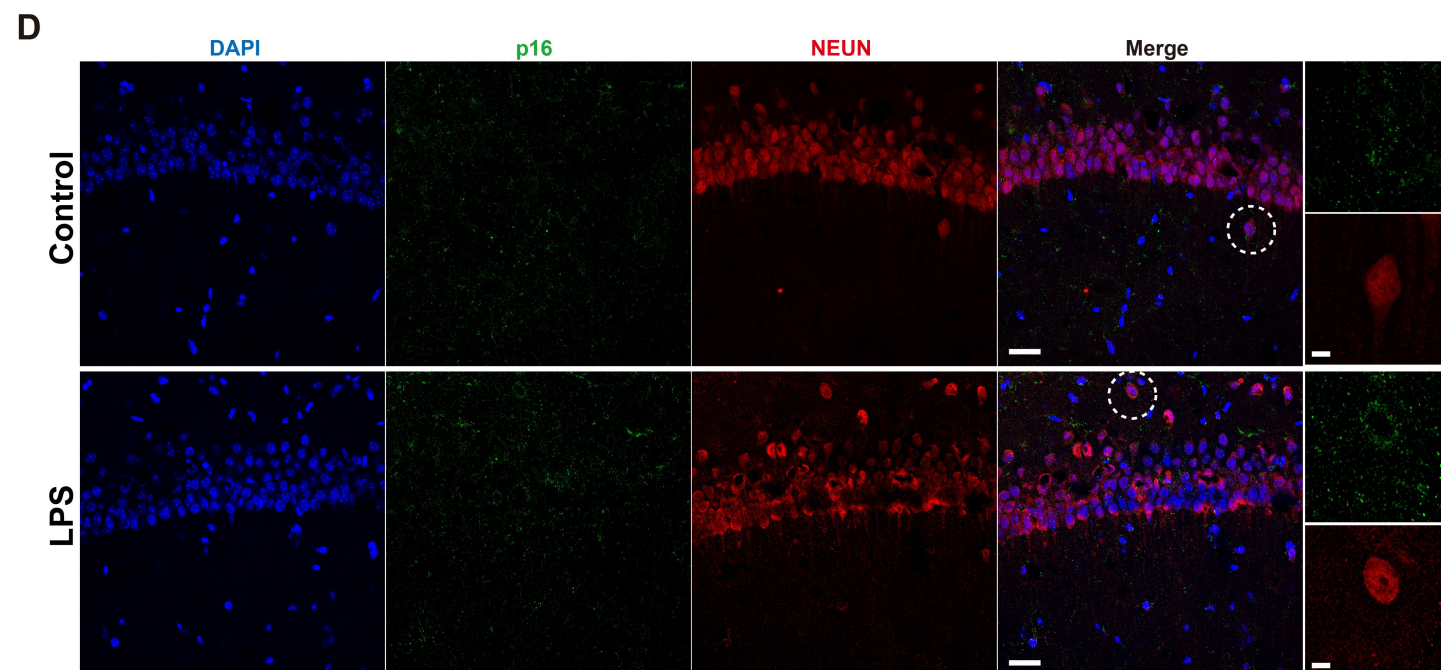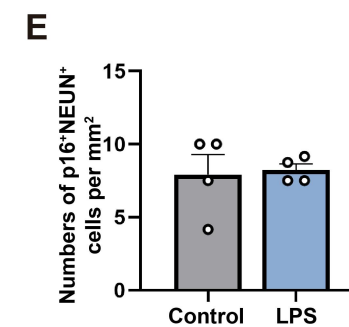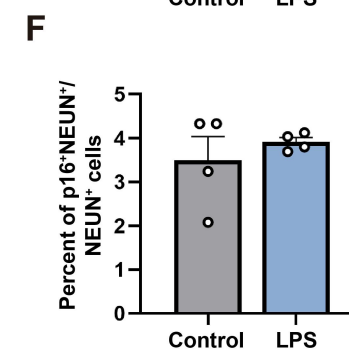

Supplement: Supplementary file 2 — Figure S2. LPS did not alter the percent of senescent neurons or astrocytes in the CA1. (A) Representative images immunostained for DAPI+ (blue), p16+ (green) and GFAP+ (red) in the hippocampal CA1 between the control and LPS groups. Scale bar = 20 μm, Zoom = 4 μm. (B) The number of p16+GFAP+ cells in the hippocampal CA1 between the control and LPS groups, N = 4 mice for each group. (C) The percent of p16+GFAP+ cells in the hippocampal CA1 between the control and LPS groups, N = 4 mice for each group. (D) Representative images immunostained for DAPI+ (blue), p16+ (green) and NEUN+ (red) in the hippocampal CA1 between the control and LPS groups. Scale bar = 20 μm, Zoom = 4 μm. (E) The number of p16+NEUN+ cells in the hippocampal CA1 between the control and LPS groups, N = 4 mice for each group. (F) The percent of p16+NEUN+ cells in the hippocampal CA1 between the control and LPS groups, N = 4 mice for each group. All values are shown as mean ± SEM by two‐tailed t test, *p < 0.05. [file ACEL-24-e70167-s004.pdf]

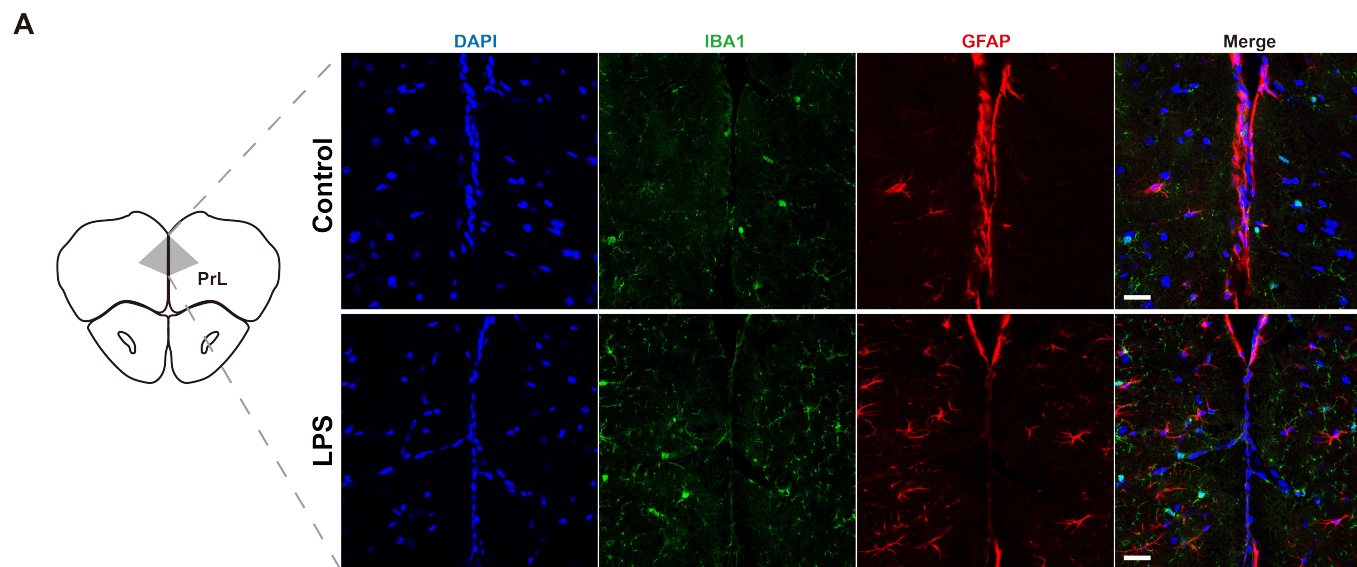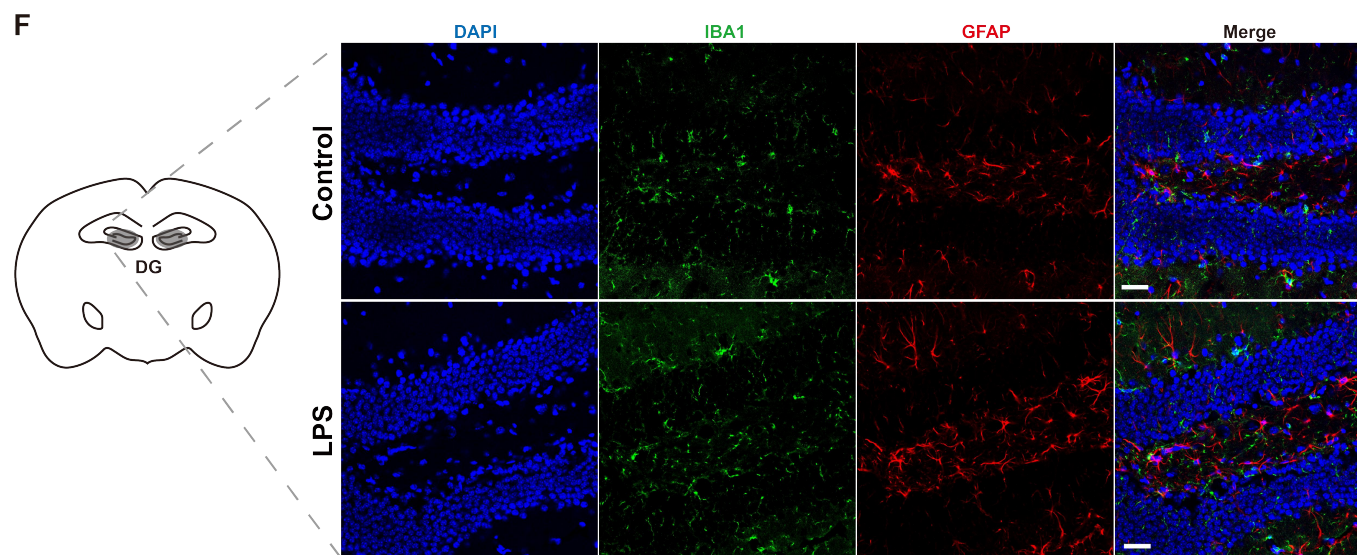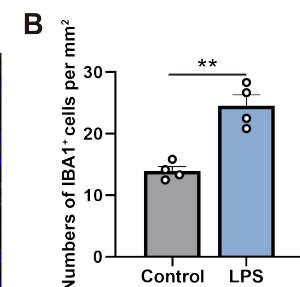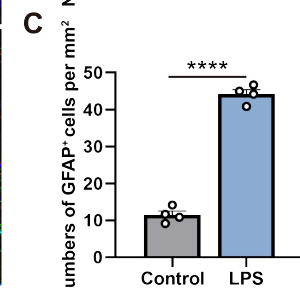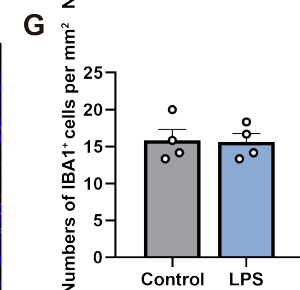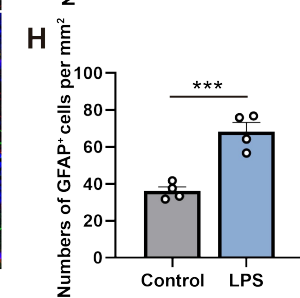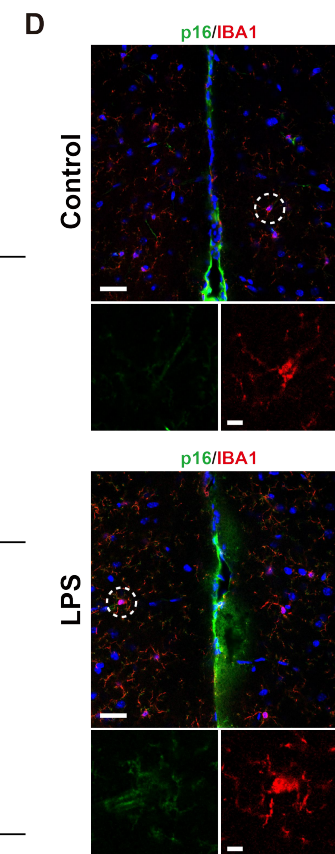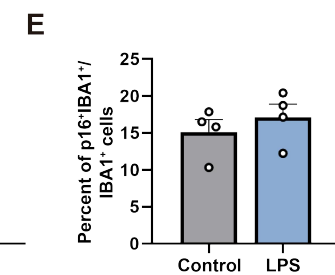

Supplement: Supplementary file 3 — Figure S3. LPS induced neuroinflammation but didn’t result in the accumulation of senescent microglia in the DG and the PrL. (A) Representative images immunostained for IBA1+ (green) and GFAP+ (red) in the PrL between the control and LPS groups. Scale bar = 20 μm. (B) The number of IBA1+ cells in the PrL between the control and LPS groups, N = 4 mice for each group. (C) The number of GFAP+ cells in the PrL between the control and LPS groups, N = 4 mice for each group. (D) Representative images immunostained for p16+ (green) and IBA1+ (red) in the PrL between the control and LPS groups. (E) Representative images immunostained for DAPI+ (blue), p16+ (green) and IBA1+ (red) in the PrL between the control and LPS groups. Scale bar = 20 μm, Zoom = 4 μm. (F) Representative images immunostained for IBA1+ (green) and GFAP+ (red) in the hippocampal DG between the control and LPS groups. Scale bar = 20 μm. (G) The number of IBA1+ cells in the hippocampal DG between the control and LPS groups, N = 4 mice for each group. (H) The number of GFAP+ cells in the hippocampal DG between the control and LPS groups, N = 4 mice for each group. All values are shown as mean ± SEM by two‐tailed t test, **p < 0.01, ***p < 0.001, ****p < 0.0001. [file ACEL-24-e70167-s001.pdf]

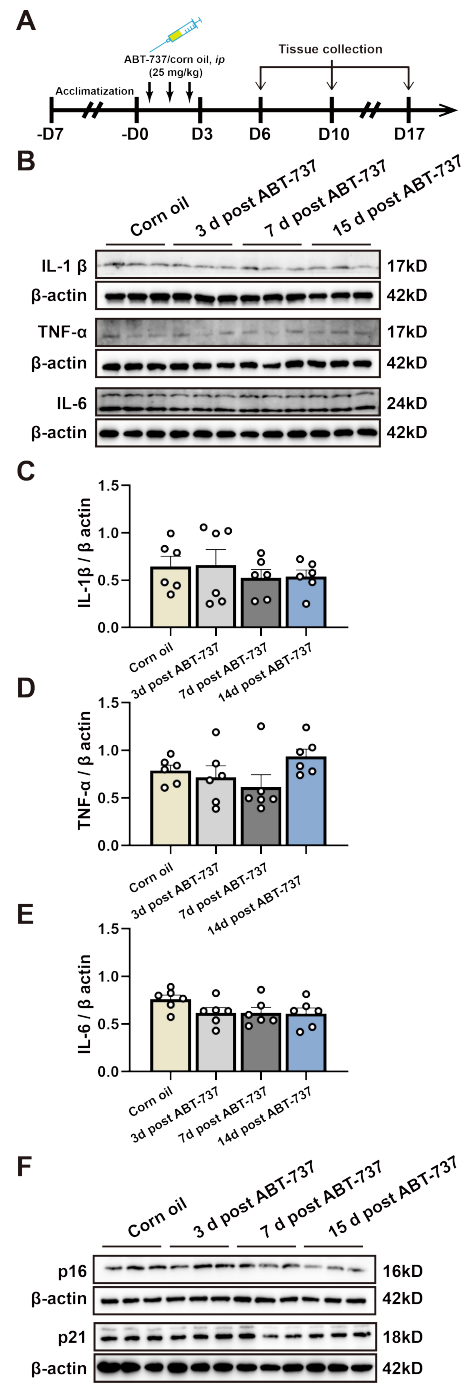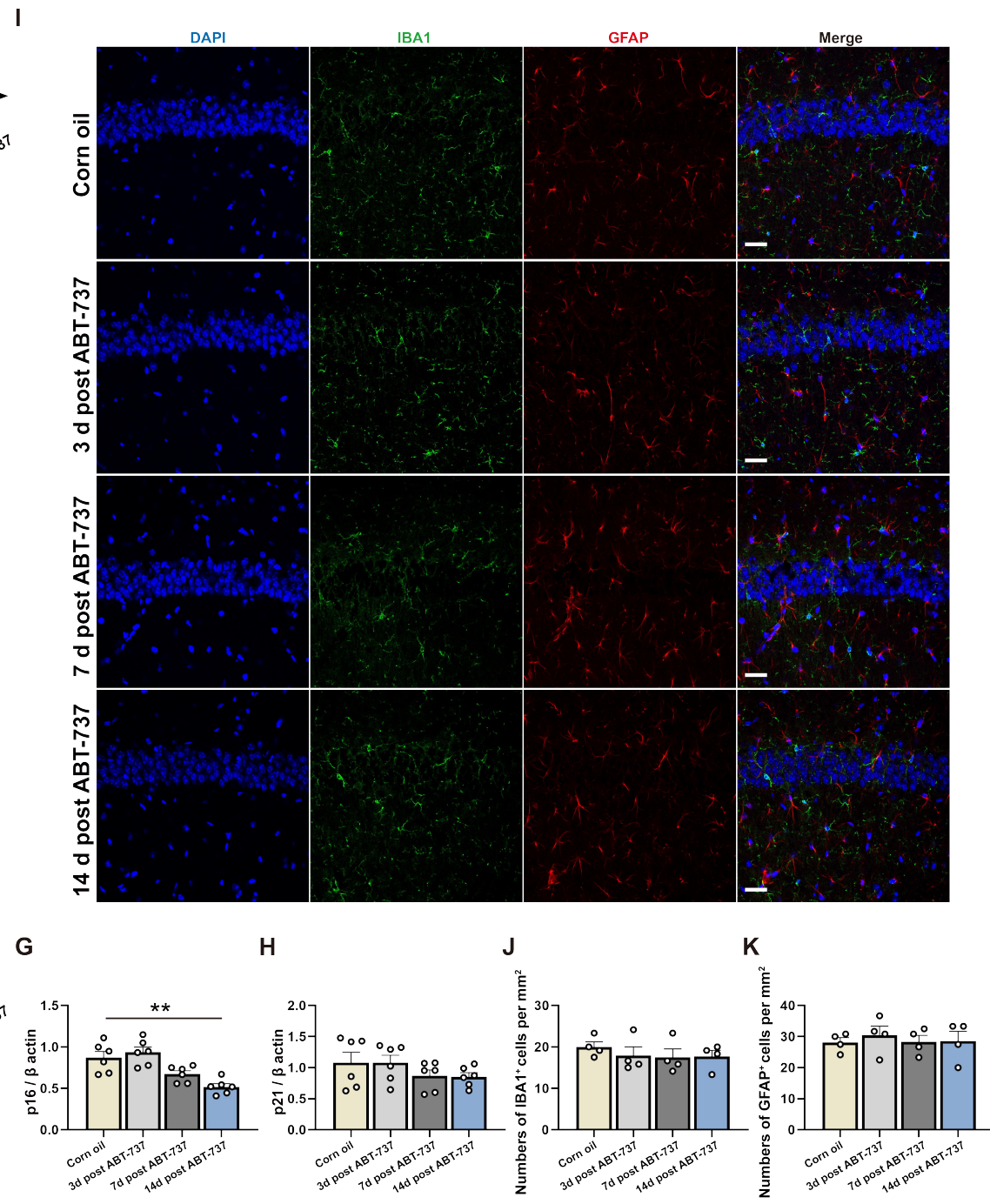

Supplement: Supplementary file 4 — Figure S4. ABT‐737 had no remarkable effect on baseline inflammatory status. (A) Schematic representation of the experimental procedures. (B) Quantification of normalized IL‐1β, TNF‐α and IL‐6 protein intensities. (C) Relative expression of IL‐1β/β actin in the hippocampi among experimental groups, N = 6 mice for each group. (D) Relative expression of TNF‐α/β actin in the hippocampi among experimental groups, N = 6 mice for each group. (E) Relative expression of IL‐6/β actin in the hippocampi among experimental groups, N = 6 mice for each group. (F) Quantification of normalized p16 and p21 protein intensities. (G) Relative expression of p16/β actin in the hippocampi among experimental groups, N = 6 mice for each group. (H) Relative expression of p21/β actin in the hippocampi among experimental groups, N = 6 mice for each group. (I) Representative images immunostained for IBA1+ (green) and GFAP+ (red) in the hippocampal CA1 among experimental groups. Scale bar = 20 μm. (J) The number of IBA1+ cells in the hippocampal CA1 among experimental groups, N = 4 mice for each group. (K) The number of GFAP+ cells in the hippocampal CA1 among experimental groups, N = 4 mice for each group. All values are shown as mean ± SEM by one‐way ANOVA with Tukey’s post hoc test, **p < 0.01. [file ACEL-24-e70167-s005.pdf]

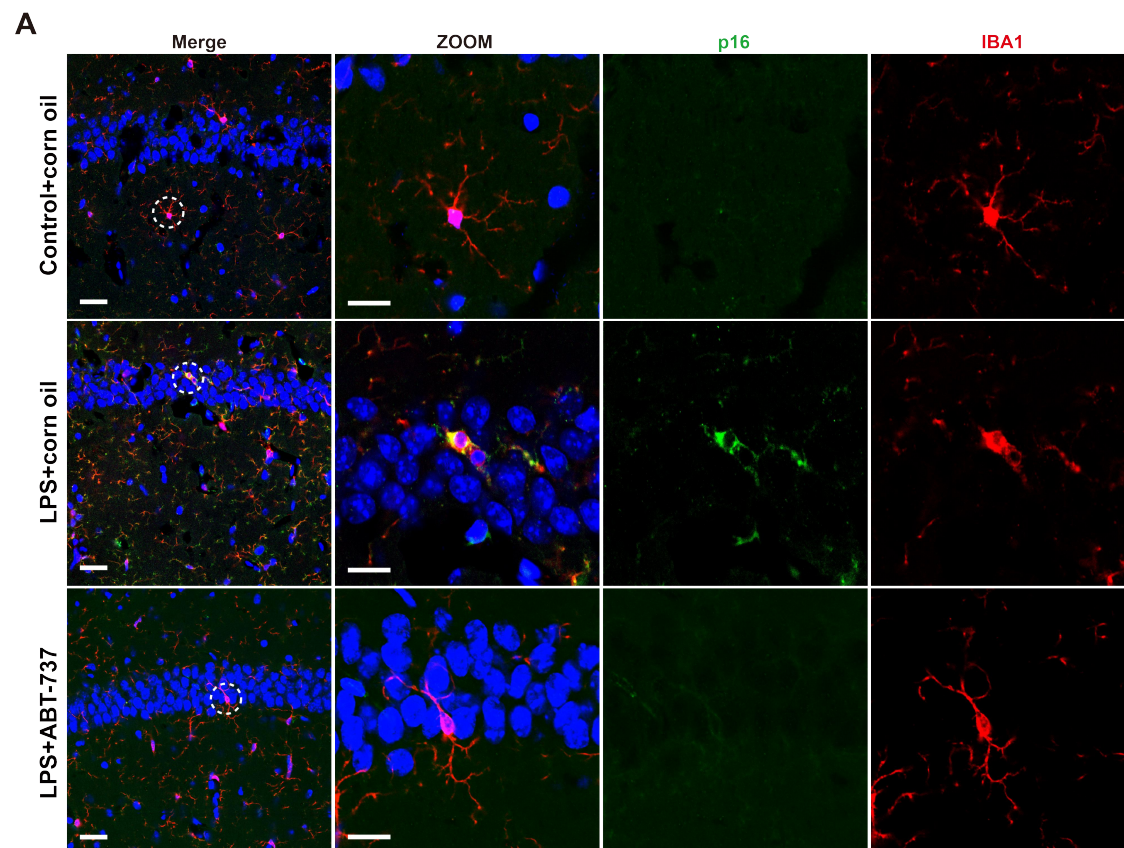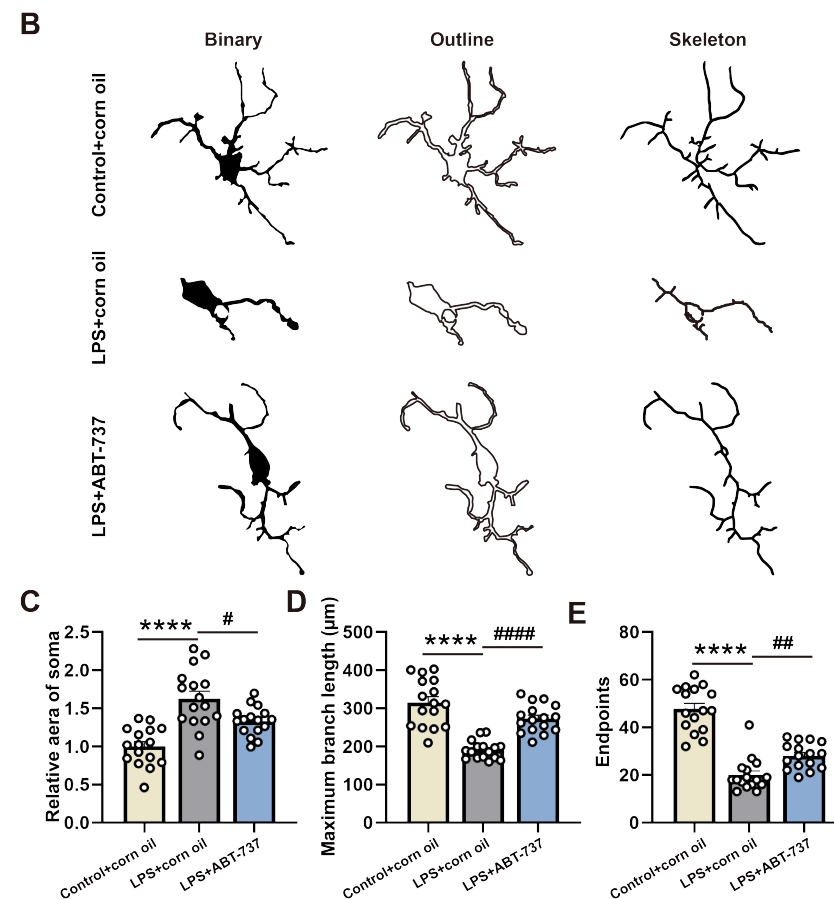

Supplement: Supplementary file 5 — Figure S5. The morphological analysis of senescent microglia in the CA1. (A) Representative images immunostained for DAPI+ (blue), p16+ (green) and IBA1+ (red) in the hippocampal CA1 among experimental groups. Scale bar = 20 μm, Zoom = 8 μm. (B) Representative binary, outlined, and skeleton images for each microglia in the hippocampal CA1 among experimental groups. (C) Relative area of soma per microglia among experimental groups, N = 16 microglia from 4 mice for each group. (D) Maximum branch length per microglia among experimental groups, N = 16 microglia from 4 mice for each group. (E) The number of endpoints per microglia among experimental groups, N = 16 microglia from 4 mice for each group. All values are shown as mean ± SEM by one‐way ANOVA with Tukey’s. [file ACEL-24-e70167-s002.pdf]
